# The complex binding mode of the peptide hormone H2 relaxin to its receptor RXFP1

**DOI:** 10.1038/ncomms11344

**Published:** 2016-04-18

**Authors:** Ashish Sethi, Shoni Bruell, Nitin Patil, Mohammed Akhter Hossain, Daniel J. Scott, Emma J. Petrie, Ross A. D. Bathgate, Paul R. Gooley

**Affiliations:** 1 Department of Biochemistry & Molecular Biology, The University of Melbourne, Victoria 3010, Australia; 2 Bio21 Molecular Science and Biotechnology Institute, The University of Melbourne, Victoria 3010, Australia; 3 Florey Institute of Neuroscience and Mental Health, The University of Melbourne, Victoria 3010, Australia; 4 School of Chemistry, The University of Melbourne, Victoria 3010, Australia

## Abstract

H2 relaxin activates the relaxin family peptide receptor-1 (RXFP1), a class A G-protein coupled receptor, by a poorly understood mechanism. The ectodomain of RXFP1 comprises an N-terminal LDLa module, essential for activation, tethered to a leucine-rich repeat (LRR) domain by a 32-residue linker. H2 relaxin is hypothesized to bind with high affinity to the LRR domain enabling the LDLa module to bind and activate the transmembrane domain of RXFP1. Here we define a relaxin-binding site on the LDLa-LRR linker, essential for the high affinity of H2 relaxin for the ectodomain of RXFP1, and show that residues within the LDLa-LRR linker are critical for receptor activation. We propose H2 relaxin binds and stabilizes a helical conformation of the LDLa-LRR linker that positions residues of both the linker and the LDLa module to bind the transmembrane domain and activate RXFP1.

## Abstract (web summary)

The mechanism by which relaxin activates the relaxin family peptide receptor-1 is poorly understood. Here, Sethi *et al.* identify a relaxin binding site in an extracellular linker between the LDLa and LRR domains and propose that relaxin binding stabilizes a helical conformation that leads to receptor activation.

## Graphical abstract

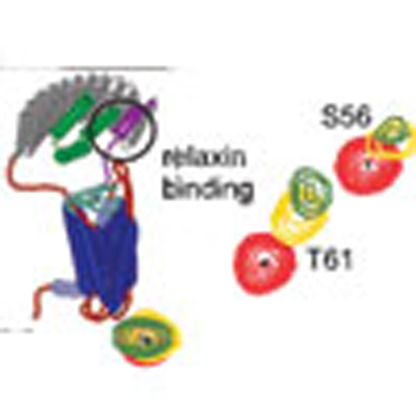

G-protein coupled receptors (GPCRs), the largest class of cell-surface receptors, are characterized by a transmembrane domain (TMD) of seven helices. Their activation mechanism typically involves the binding of an extracellular signalling molecule causing conformational changes within the TMD that trigger the coupling of G-proteins to activate downstream signalling^1^. The relaxin family peptide receptor-1 (RXFP1), is a class A GPCR that in addition to the TMD, comprises a large extracellular domain of leucine-rich repeats (LRRs) and an N-terminal low density lipoprotein class A (LDLa) module, which further classifies this receptor as a Type C leucine-rich repeat containing GPCR (LGR; Fig. 1)^2^,^3^. Only RXFP1 and the closely related INSL3 receptor, RXFP2, belong to this classification and they are the only type C LGRs found in mammals. Currently no other human GPCR containing LDLa modules has been reported^4^.

H2 relaxin, a two-chain 6-kDa peptide hormone that is structurally related to insulin, activates RXFP1. Initially characterized as a reproductive hormone with its pregnancy-associated actions^5^, H2 relaxin shows other diverse physiological actions including increasing lung perfusion and gas exchange, acting as a potent vasodilator, and showing anti-fibrotic and cardioprotective effects^6^. Due to these latter actions it has received clinical interest as a treatment for acute heart failure^7^. The clinical potential for targeting the actions of H2 relaxin has intensified research in developing relaxin-mimetic drug-like molecules with increased bioavailability and sustained action. To be successful in designing such a molecule, in-depth understanding of the relaxin/RXFP1 mechanism of activation is essential.

Activation of RXFP1 is a complex multistep process. Previous studies have demonstrated that Arg13, Arg17 and Ile20, of the arginine cassette (RxxxRxxI/V, where x is any residue) of the H2 relaxin B-chain, bind to Asp231, Asp279, Glu233 and Glu277 located on LRR4–8 of the LRR domain of RXFP1 (refs 8, 9). Importantly ligand binding alone cannot activate the receptor, rather it is the LDLa module that is essential for activation. Truncation or substitution of the LDLa module does not affect ligand binding but results in an inactive receptor^10^,^11^. The LDLa module is well-folded with three conserved disulfide bonds and a consensus motif (DxxxDxxDxxDE), which ligates Ca^2+^ that is necessary for maintaining structure^12^,^13^. Site-directed mutagenesis of the module suggests Leu7, Tyr9 and Lys17 in the N-terminal portion of the module are involved in receptor activation^10^,^12^. In a recent study^14^, both the LDLa module and H2 relaxin were shown to interact with exoloops-1 and -2 (EL1 and EL2) of the TMD when grafted onto an engineered scaffold protein^15^. These observations support that the LDLa module is the true ligand that interacts with the TMD to cause conformational rearrangement and G-protein coupling, although this mechanism requires H2 relaxin binding. This is clearly a unique mode of GPCR activation, where the ligand itself is not the activator but drives a receptor-attached module to initiate downstream signalling.

The LDLa module of RXFP1 is joined to the LRR domain via a 32-residue linker. Except for a region (GDNNGW) immediately C-terminal to the LDLa module the linker appears poorly conserved and superficially is a simple unstructured spacer (Fig. 1). A recent study swapped the LDLa modules of RXFP1 and 2, and found that the correct length of the linker was necessary for activation, suggesting that the linker has additional functionality^16^. The present study shows that the linker does contain residues essential for receptor activation. While the LDLa-LRR linker appears intrinsically unstructured there is a region of residual structure that contains a binding site for H2 relaxin, which in combination with the binding site of the LRR domain, is required for the nanomolar affinity of H2 relaxin for its receptor. Finally, we demonstrate that the LDLa-LRR linker may interact with the exoloops of the TMD supporting the critical role of this region in receptor activation.

## Results

### Investigating the role of the RXFP1 LDLa-LRR linker

**Figure 2:**
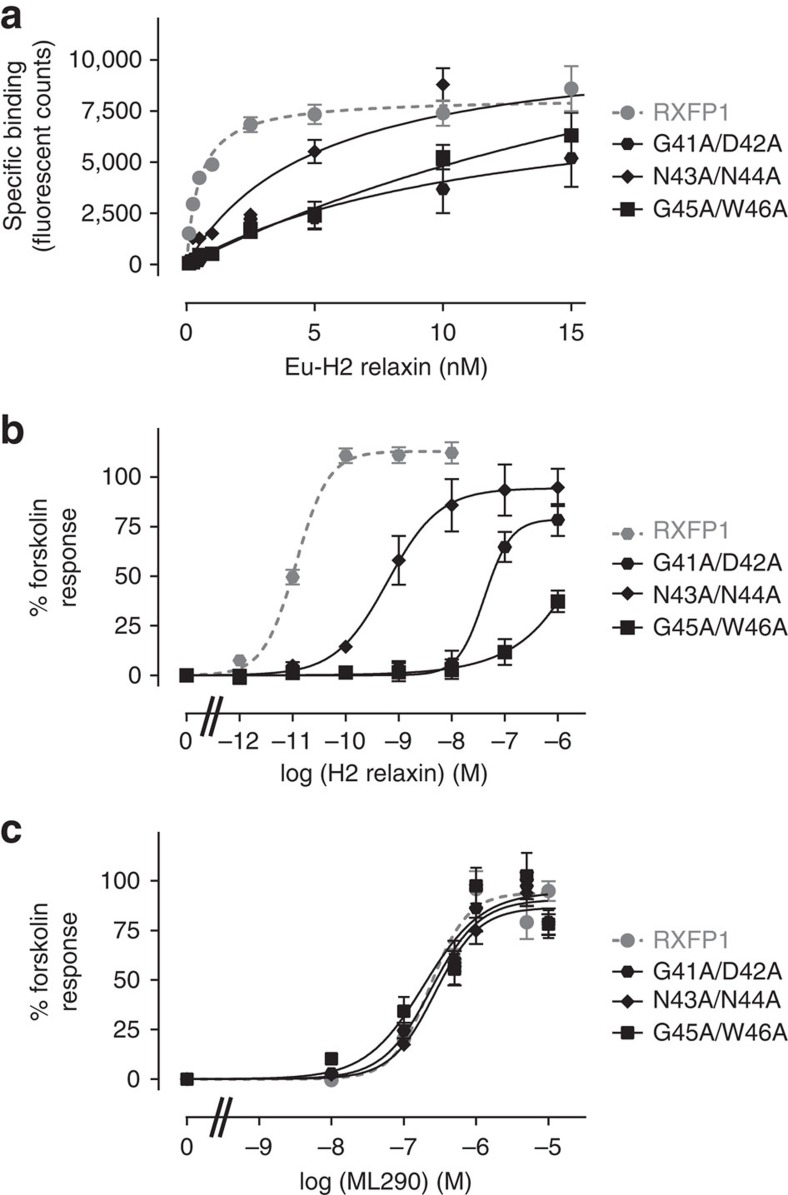
H2 relaxin binding and activation of wild type and LDLa-linker mutants of RXFP1. (**a**) Saturation binding using Eu-H2 relaxin. (**b**) H2 relaxin-induced cAMP responses. (**c**) ML290-induced cAMP responses. Symbols represent mean values±s.e.m. from triplicate values in a minimum of three independent experiments.

While the LDLa module of RXFP1 is indispensable for receptor activation^10^ and the LRR domain is considered as the primary H2 relaxin-binding site^8^,^9^, the role of the 32 residues linking the two domains has not been investigated. Here, we used transiently transfected human embryonic kidney (HEK) 293T cells expressing full-length RXFP1 and mutants to assess H2 relaxin binding and induced cAMP activation. Activation of these receptors was further tested with the small molecule RXFP1 agonist (ML290) that binds directly to the TMD activating RXFP1 in an allosteric and H2 relaxin-independent manner^17^. Initially we made three double mutants, G41A/D42A, N43A/N44A and G45A/W46A. These mutant receptors expressed at the cell surface and responded to ML290 activation comparable to wild-type receptor (Fig. 2c, Table 1) indicating that mutation of linker residues does not affect receptor trafficking or G-protein coupling. However, mutation of these residues had a profound effect on both H2 relaxin binding and activation. N43A/N44A demonstrated a fivefold loss of binding affinity for H2 relaxin, while G41A/D42A and G45A/W46A bound 25-fold more weakly (Fig. 2a, Table 1). Surprisingly the mutant receptors demonstrated reduced H2 relaxin potency, which was far greater than the reduction in affinity. N43A/N44A and G41A/D42A showed 500- and 5,000-fold loss of H2 relaxin-stimulated cAMP activity while G45A/W46A was virtually unable to signal (Fig. 2b, Table 1).

We then investigated the contribution of the individual amino acids in this region to both H2 relaxin binding and activation. Mutation of Asp42 and Asn43, but not Gly41 and Asn44, clearly contribute to H2 relaxin binding and activation (Table 1). Mutating Gly45 or Trp46 caused reductions in binding and activation, but less than the combined mutant (G45A/W46A) suggesting both residues contribute to H2 relaxin binding and activation. Collectively these data suggest residues of the linker are important in both ligand binding and receptor activation.

### Mapping the H2 relaxin-binding site on RXFP1_(1–72)_

**Figure 3:**
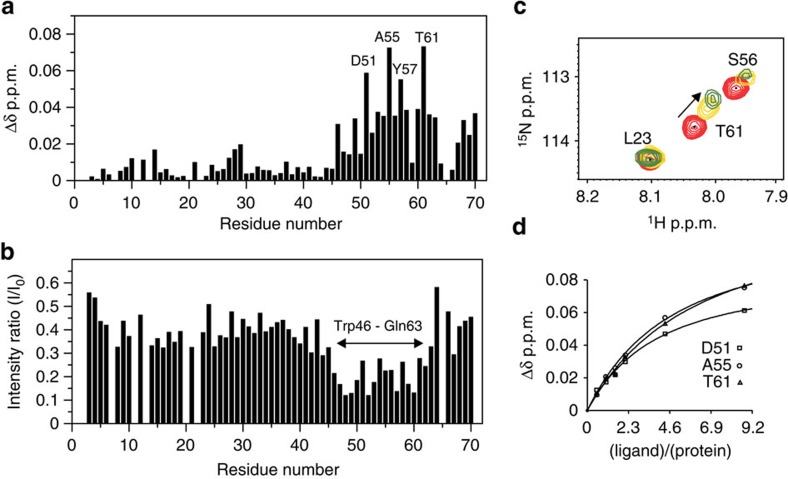
Titration of 50 μM ^15^N-labelled RXFP1_(1–72)_ with H2 relaxin. (**a**) Plot of the change in average ^1^HN and ^15^N chemical shifts and (**b**) peak intensities following titration of ^15^N-RXFP1_(1–72)_ with nine equivalents of H2 relaxin. (**c**) Representative region of the ^1^H,^15^N HSQC spectrum showing chemical shift dependence on H2 relaxin. (**d**) Single-site saturation binding curves (*K*_d_=200±10 μM) for the three resonances that show the largest chemical shift changes and remained resolved throughout the titration. Experiments were conducted at pH 6.8 and 25 °C.

The weakening of H2 relaxin binding by mutations within the region GDNNGW was unexpected. To gain detailed insight into the role of the linker in H2 relaxin binding, we recombinantly expressed and purified fractionally deuterated ^13^C,^15^N-labelled LDLa module with the 32 residues of the linker, designated RXFP1_(1–72)_ and assigned all the backbone resonances (Supplementary Fig. 1). To investigate the interaction between RXFP1_(1–72)_ and H2 relaxin, we performed a two-dimensional (2D) ^1^H-^15^N HSQC monitored H2 relaxin titration of ^15^N-labelled RXFP1_(1–72)_. Significant chemical shift and intensity differences were noted for residues assigned to the linker region comprising Trp46 to Gln63 (Fig. 3a,b). From the chemical shift difference plot, Asp51, Ala55, Tyr57 and Thr61 (Fig. 3a,c) experienced the largest chemical shift changes. Fitting these differences of Asp51, Ala55 and Thr61, which remain resolved in the ^1^H-^15^N HSQC spectra throughout the titration, to a single-site binding curve shows the affinity of H2 relaxin for RXFP1_(1–72)_ is 200±10 μM (Fig. 3d). Importantly, residues within the LDLa module are largely unperturbed and residues from Gly41 to Asn45 also show minimal chemical shift and intensity changes, suggesting that although mutation to this region perturbs activity it does not directly bind H2 relaxin.

### Probing binding to RXFP1_(1–72)_ with Mn^2+^-labelled H2 relaxin

**Figure 4:**
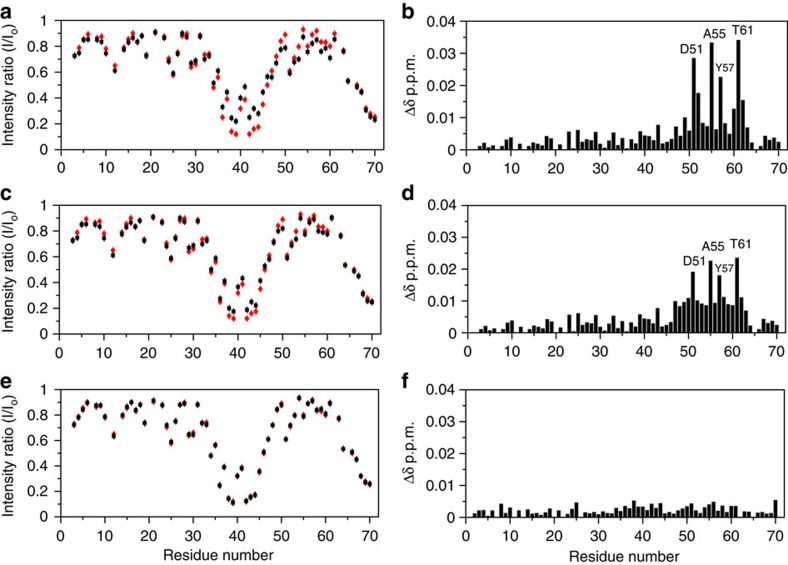
Plots of intensity ratios and chemical shift changes of ^15^N-labelled RXFP1_(1–72)_ titrated with H2 relaxin or analogues. (**a**,**b**) H2 relaxin, (**c**,**d**) A-chain (9–24) H2 relaxin and (**e**,**f**) H2/I5-2 H2 chimera. In (**a**,**c**,**e**) (red circles) represent 50 μM ^15^N-labelled RXFP1_(1–72)_ in the presence of 0.2 μM Mn^2+^-DTPA-(A)-H2; (black squares) indicate changes to the intensity ratio following addition of one molar equivalent H2 relaxin or analogue. (**b**,**d**,**f**) show the change in average ^1^HN and ^15^N chemical shifts after titration of the RXFP1_(1–72)_ and Mn^2+^-DTPA-(A)-H2 complex with one molar equivalent of H2 relaxin or analogue. Experiments were conducted at pH 6.8 and 25 °C. Error bars represent the average estimated experimental noise for the respective NMR experiment.

H2 relaxin has a propensity to dimerize at high concentrations^18^, therefore we probed the interaction of RXFP1_(1–72)_ with substoichiometric concentrations of paramagnetically labelled H2 relaxin. As previously demonstrated^19^, the use of a diethylene triamine pentaacetic acid (DTPA) cage attached to the N-terminus of the A-chain of H2 relaxin does not perturb binding to RXFP1 when loaded with Eu^3+^. Replacement of Eu^3+^ with Mn^2+^ (Mn^2+^-DTPA-(A)-H2) showed no difference on binding to or activating RXFP1. Therefore we monitored paramagnetic (Mn^2+^) induced line-broadening in ^1^H-^15^N HSQC by titrating 50 μM ^15^N-labelled RXFP1_(1–72)_ with Mn^2+^-DTPA-(A)-H2 (0–0.3 μM). The most significant effects were localized to Asp36 to Trp46 which comprise the C-terminus of the LDLa module and the first six residues of the linker (Fig. 4a). Considering the paramagnetic radius of Mn^2+^, broadening of nuclei can be up to 34 Å from the paramagnetic probe^20^. The observed broadening is localized and specific, suggesting that this region is in close proximity to the DTPA cage attached at the N-terminus of the A-chain. Importantly, the resonances from Asp51 to Thr61, which show the largest chemical shift changes on H2 relaxin titration, were not broadened (Figs 3a and 4a) indicating that these residues are distant from the cage and the A-chain N-terminus.

To further identify the specificity of the Mn^2+^-DTPA-(A)-H2 interaction with the linker residues, we performed a competition titration with H2 relaxin (up to 50 μM) against 50 μM ^15^N-labelled RXFP1_(1–72)_ mixed with 0.2 μM Mn^2+^-DTPA-(A)-H2. The specificity of the interaction with Mn^2+^-DTPA-(A)-H2 was confirmed by the reappearance of the broadened peaks in the region Asp36–Trp46 on addition of native H2 relaxin (Fig. 4a and Supplementary Fig. 2). We also detected similar chemical shift changes and resonance broadening of the region Trp46–Gln63 on addition of H2 relaxin, equivalent to the initial H2 relaxin titration (Figs 3a and 4b and Supplementary Fig. 2). While confirming the specificity of the interaction between the LDLa-LRR linker with H2 relaxin, these experiments also contribute to understanding the orientation of H2 relaxin binding. As the DTPA cage is at the N-terminus of the A-chain of H2 relaxin, and previous studies have mapped the binding of the arginine cassette on the B-chain to the LRR domain^8^,^9^, it is likely that interaction with the LDLa-LRR linker is mediated via the A-chain.

### Residues of the A-chain of H2 relaxin bind RXFP1_(1–72)_

To further probe the role of the H2 relaxin A-chain in the linker interaction, we performed competition titrations with various truncated and chimeric H2 relaxin peptides against the complex of 50 μM ^15^N-labelled RXFP1_(1–72)_ with 0.2 μM Mn^2+^-DTPA-(A)-H2. First, we tested an N-terminally truncated A-chain (9–24) H2 peptide, which shows decreased affinity and potency for RXFP1 (ref. 21). On addition of 50 μM of this peptide to the RXFP1_(1–72)_/Mn^2+^-DTPA-(A)-H2 complex, previously broadened peaks reappeared (Fig. 4c); however, the gain in peak intensity was not as pronounced as that recorded with native H2 peptide (Fig. 4a). Similarly, the chemical shift perturbations detected in the linker residues, while consistent with native H2 competition, were significantly lower in magnitude (Fig. 4d). These results suggest that the N-terminal truncation in the A-chain of H2 relaxin perturbs affinity for the linker, and is consistent with decreased binding to full-length RXFP1 (ref. 21).

Next we tested an A-chain H2 chimeric analogue (H2/I5-2) (ref. 22). This analogue differs to H2 relaxin where the middle three residues between the two cysteines of the A-chain are replaced with those of the A-chain of INSL5, which differs in sequence by two residues, His12 and Val13 (H2) to Thr12 and Asp13 (INSL5). This chimera shows a modest loss of binding, but retains the ability to activate RXFP1 (ref. 22). On addition of 50 μM H2/I5-2 to the RXFP1_(1–72)_/Mn^2+^-DTPA-(A)-H2 complex no changes were observed in peak intensities or chemical shifts (Fig. 4e,f) suggesting that His12 and Val13 are essential to the interaction of H2 relaxin with the linker.

We then performed a competition experiment with the H2 relaxin mutant F23A (ref. 22). This mutation destabilizes the secondary structure of the A-chain, but not the B-chain, resulting in significant loss of binding affinity and activation of RXFP1 (ref. 22). This mutant peptide did not compete with Mn^2+^-DTPA-(A)-H2 and did not induce any chemical shift perturbation in the linker residues (Supplementary Fig. 3c,d). We also tested truncations in the B-chain of H2 relaxin on binding to RXFP1_(1–72)_. We selected two B-chain truncates, H2-(B1–25) (Supplementary Fig. 3e,f) and H2-(B5–29) (Supplementary Fig. 3g,h), to investigate the role of N- and C-terminal residues of the B-chain in binding to RXFP1_(1–72)_. Previous studies have shown that these truncated peptides retain their native affinity and potency at RXFP1 (ref. 23). For both peptides, we did not detect any significant difference in binding to RXFP1_(1–72)_ as demonstrated by their ability to compete with Mn^2+^-DTPA-(A)-H2 peptide. Taken together the competition experiments demonstrate that the A-chain of H2 relaxin and the preservation of its secondary structure are critical for the interaction with the LDLa-LRR linker.

### Residual helical structure in the LDLa-LRR linker

**Figure 5:**
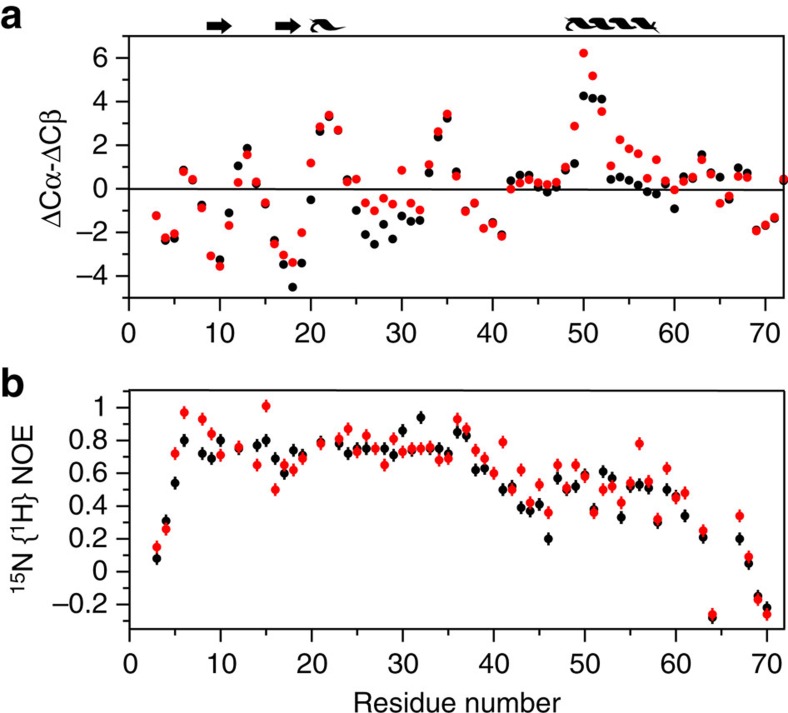
Plots of ^13^Cαβ secondary chemical shifts and ^15^N{^1^H}-NOEs of RXFP1_(1–72)_ in the absence (black) and presence (red) of three molar equivalents of H2 relaxin. (**a**) Deviations of the measured ^13^Cα and ^13^Cβ chemical shifts from random coil where persistent positive or negative deviations indicate the presence of α-helix or β-strand, respectively. Above this plot is schematically represented the known secondary structure of the LDLa module and included a region of α-helix that increases in the presence of H2 relaxin to spanning Leu48 to Ser56. (**b**) Plot of ^15^N{^1^H}-NOEs that shows on addition of relaxin the ^15^N{^1^H}-NOEs increase for the region Gly41 to Lys59. Experiments were conducted at pH 6.8 and 25 °C. Error bars represent the average estimated experimental noise for the respective NMR experiment.

^15^N and ^13^C-edited NOESY spectra do not show any stable structure within the linker region for RXFP1_(1–72)_. However, ^13^Cα and ^13^Cβ are sensitive to the presence of secondary structure in proteins, including intrinsically unstructured proteins^24^,^25^. Indeed for RXFP1_(1–72)_ we observe the persistence of positive and negative (ΔCα—ΔCβ) smoothed values^26^ consistent with the expected helical and β-strand structure of the LDLa module^12^. In the absence of H2 relaxin, the (ΔCα—ΔCβ) smoothed values suggest that the linker is largely unstructured, except for a region of positive values that point to the presence of a turn of α-helix comprising residues Gln49 to Lys52 (Fig. 5a). On titration of ^13^C,^15^N-labelled RXFP1_(1–72)_ with H2 relaxin the helical propensity of these residues increases and extends to include Leu48 to Ser56 suggesting H2 relaxin binds and stabilizes a helical structure in the linker. To further characterize the presence of residual structure, we recorded ^15^N{^1^H}-NOE experiments^27^ on ^15^N-labelled RXFP1_(1–72)_ (Fig. 5b). The ^15^N{^1^H}-NOE values (0.74±0.08) for the LDLa module (residues 6–40) agree with a folded structure (PDB 2jm4), with a flexible N-terminus (residues 1–5, ^15^N{^1^H}-NOEs <0.4). In the linker region, a progressive decrease in ^15^N{^1^H}-NOE to 0.2 is observed for Gly41 to Trp46, suggesting increasing flexibility. However, this decrease is followed by a rise in the ^15^N{^1^H}-NOEs to 0.49±0.10 (Leu48 to Met60) consistent with the presence of residual structure across the relaxin-binding site. Following Thr61 the ^15^N{^1^H}-NOE progressively decreases, implying a flexible C-terminal region. On addition of relaxin to ^15^N-labelled RXFP1_(1–72)_, little change is observed in the ^15^N{^1^H}-NOEs for residues 6–40 of the LDLa module (0.75±0.10). However, for the linker region from Gly41 to Lys59 there is a distinct increase in ^15^N{^1^H}-NOEs, from 0.47±0.11 without H2 relaxin to 0.55±0.13 with H2 relaxin, supporting stabilization of helical structure within this region.

### Role of LDLa-LRR linker residues in H2-mediated activation

The NMR experiments highlighted a significant and specific interaction between the A-chain of H2 relaxin and RXFP1_(1–72)_ suggesting that residues within the region Trp46 to Gln63 bind H2 relaxin (Fig. 3). Guided by chemical shift changes and the stabilization of helix on H2 relaxin titration we investigated the functional effect of mutating residues on full-length RXFP1 (Table 1). All of the mutant receptors expressed at the cell surface similar to wild-type RXFP1. Of the single-site mutations F50A, F54A and Y58A showed significantly reduced binding affinity for H2 relaxin (Table 1, Supplementary Fig. 4a). F50A and F54A, but not Y58A, showed significant reduction in H2-mediated activation (Table 1, Supplementary Fig. 4b). Mutations near these residues, S47A/L48A, D51A, A55S and Y57A, showed no significant difference in H2 relaxin affinity or induced activation (Table 1). The mutant A55L, however, showed a modest reduction in affinity and activation. Compared to the single-site mutations, the combined mutant F54A/Y58A showed a compounded effect on H2 relaxin affinity and a further reduction in H2-mediated activation (Table 1, Supplementary Fig. 4). All these mutants showed no change to relaxin-independent ML290 activation (Table 1, Supplementary Fig. 4c).

We also made a series of insertion and deletion mutants to investigate the effect of the length of the LDLa-LRR linker on receptor function. Insertion of an Ala residue before GDNNGW (C40A^ins^) significantly weakened H2 relaxin affinity and completely abolished H2-mediated activation (Table 1, Supplementary Fig. 5a,b). The insertion mutant, F50A^Ins^ showed a significant decrease in affinity and reduced activation. Notably both mutants showed no changes in ML290-mediated activation (Table 1, Supplementary Fig. 5c). Insertion (E67A^Ins^) or deletion (A68^Δ^) at the end of the linker showed no significant change in H2-mediated activation (Table 1). As the insertions made at the end of the linker did not perturb receptor function, it appears that the Ala insertions after Cys40 and Phe50 are specific to this region of the linker, rather than a non-specific effect of lengthening the linker.

### Structural effect of mutations on the LDLa-LRR linker

**Figure 6:**
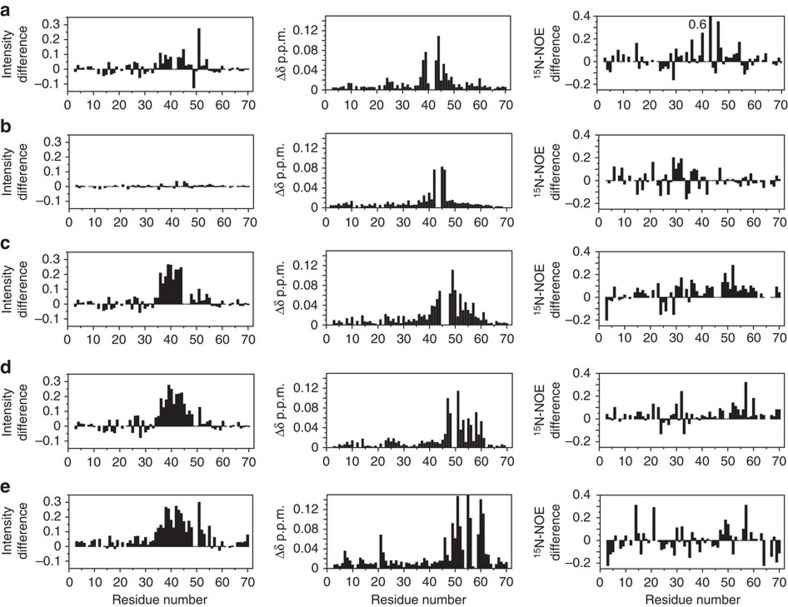
Comparison of binding of Mn^2+^-DTPA-(A)-H2, chemical shift differences and ^15^N{^1^H}-NOEs of wild type and mutants of RXFP_(1–72)_. The first column is the difference (mutant less wild type) in peak intensities from a titration of 50 μM mutant and wild-type LDLa-linker with 0.2 μM Mn^2+^-DTPA-(A)-H2. A positive difference indicates less binding of Mn^2+^-DTPA-(A)-H2 to the mutants. The second column is the average ^1^HN and ^15^N chemical shift differences (Δδ) of mutant to wild-type protein. The third column is the difference (wild-type less mutant) of ^15^N{^1^H}-NOE of mutant and wild-type LDLa-linker. A positive difference indicates a lower ^15^N{^1^H}-NOE in the mutant. Experiments were conducted at pH 6.8 and 25 °C on (**a**) G41A/D42A, (**b**) N43A/N44A, (**c**) G45A/W46A, (**d**) F50A, and (**e**) F54A/Y58A. Additional mutants and data are in Supplementary Fig. 6.

The site-directed mutagenesis experiments on RXFP1 suggested that the first six residues within the linker (GDNNGW) are functionally important. The results from the NMR experiments conducted on RXFP1_(1–72)_, however, suggested that these residues are not directly involved in side-chain interactions with H2 relaxin. To further understand the role of individual linker residues in the interaction with H2 relaxin we translated the mutations that had the most profound effect on RXFP1 activity and H2 relaxin binding to the recombinant RXFP1_(1–72)_ (C40A^ins^, G41A/D42A, N43A/N44A, G45A/W46A, F50A, F50A^Ins^, F54A and F54A/Y58A). We assessed the ^15^N and ^1^HN chemical shift effects of mutation, differences in their ^15^N{^1^H}-NOE profile and H2 relaxin binding by titration with Mn^2+^-DTPA-(A)-H2 (Fig. 6, Supplementary Fig. 6).

Titration of G45A/W46A-RXFP1_(1–72)_ with Mn^2+^-DTPA-(A)-H2 broadened resonances of residues within Asp36 to Trp46, but substantially less than that observed for wild-type RXFP1_(1–72)_ (Fig. 6c, Supplementary Fig. 6d). Importantly, this mutant compared with wild-type RXFP1_(1–72)_ showed extensive chemical shift changes to resonances encompassing Gly41 to Tyr57, which includes our proposed H2 relaxin-binding site, and lower ^15^N{^1^H}-NOEs particularly from Leu48 to Met60, but also the C-terminal region of the LDLa module, Asp30 to Cys40 (Fig. 6c). These data suggest that while Gly45 and Trp46 may interact with H2 relaxin they are important for maintaining the structure of the binding epitope within the LDLa-LRR linker and its disruption partly accounts for the >30-fold loss of H2 relaxin affinity for G45A/W46A-RXFP1 (Table 1). Line-broadening by Mn^2+^-DTPA-(A)-H2 was attenuated for G41A/D42A-RXFP1_(1–72)_, but less than that observed for G45A/W46A-RXFP1_(1–72)_ (Fig. 6a, Supplementary Fig. 6b). Except for the region of mutation, the ^15^N{^1^H}-NOEs for G41A/D42A-RXFP1_(1–72)_ are similar to wild-type RXFP1_(1–72)_. Significant chemical shift differences were observed for resonances of residues spanning Asp38 to Gln49, but these are mostly outside the H2 relaxin-binding epitope (Fig. 6a and Fig. 2). Therefore the >30-fold loss of H2 relaxin affinity by G41A/D42A-RXFP1 may include additional factors other than maintaining the H2 relaxin-binding site of the linker. N43A/N44A- and C40A^ins^-RXFP1_(1–72)_ bound Mn^2+^-DTPA-(A)-H2 similar to wild-type. These mutants also showed no differences in the ^15^N{^1^H}-NOEs within the linker (Fig. 6b; Supplementary Fig. 6) compared with wild-type and chemical shift differences were restricted to near the sites of mutation, thereby suggesting that the H2 relaxin-binding epitope in the LDLa-LRR linker is maintained. As these mutations in full-length RXFP1 showed ∼sixfold decrease in H2 relaxin affinity (Table 1), additional factors must account for these losses.

Titration of F54A-RXFP1_(1–72)_ with Mn^2+^-DTPA-(A)-H2 broadens resonances of residues, but less than that observed for wild-type RXFP1_(1–72)_ (Supplementary Fig. 6g), consistent with the fourfold loss in H2 relaxin binding to F54A-RXFP1 (Table 1). F54A-RXFP1_(1–72)_ mutation minimally perturbs resonances (Δδ>0.025 p.p.m.) distant from the mutation site. Furthermore, the ^15^N{^1^H}-NOEs of the H2 relaxin-binding region (Leu48 to Met60) of F54A-RXFP1_(1–72)_ are similar to those of wild type suggesting that the side chain of Phe54 directly contacts H2 relaxin. F50A and F54A/Y58A show similar effects to each other. In both of these mutants, broadening by Mn^2+^-DTPA-(A)-H2 is substantially reduced (Fig. 6d,e), and compared with F54A a wide range of resonances, Trp46 to Met60, are affected. These findings are consistent with both mutants showing a tenfold loss in H2 relaxin binding to the equivalent mutations in RXFP1 (Table 1), but the extent of chemical shift changes suggest that more than a simple epitope of side chains has been removed. Indeed the ^15^N{^1^H}-NOEs for the region Leu48 to Met60, similar to G45A/W46A-RXFP1_(1–72)_, decrease compared with wild-type RXFP1_(1–72)_, suggesting a loss of structure. The insertion mutant F50A^Ins^-RXFP1_(1–72)_ also showed extensive chemical shift differences (Trp46 to Tyr57), reduced peak broadening with Mn^2+^-DTPA-(A)-H2 and decreased ^15^N{^1^H}-NOEs (Supplementary Fig. 6f). For this mutant, these changes are generally less marked than those observed for F50A and F54A/Y58A; however, the loss of binding to H2 relaxin by F50A^Ins^-RXFP1 is 25-fold (Table 1) which is greater than that observed for F50A, F54A and F54A/Y58A suggesting additional factors for binding of H2 relaxin in full-length RXFP1 have been perturbed.

### LDLa-LRR linker interactions with the TMD exoloops

**Figure 7:**
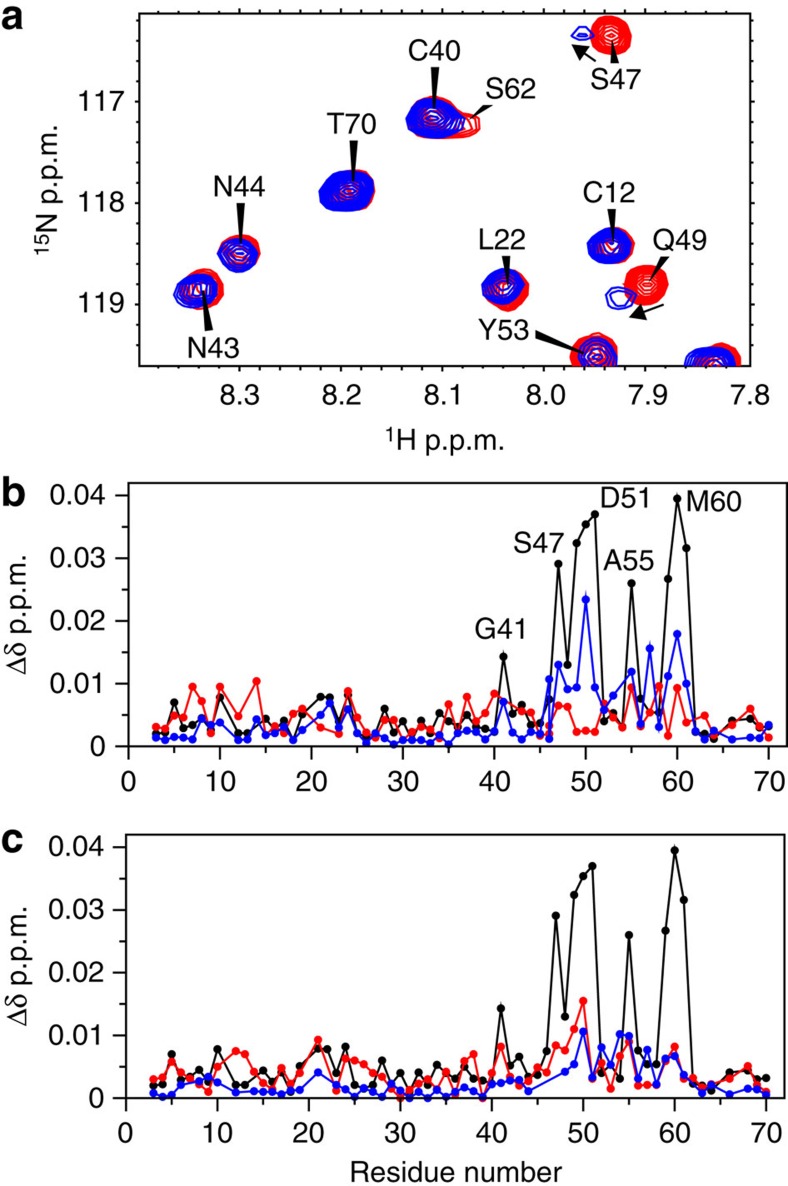
Chemical shift differences following a titration of ^15^N-labelled RXFP1_(1–72)_ with 20 molar equivalents of EL1^(475–486)^/EL2-RXFP1. (**a**) portion of ^1^H-^15^N HSQC spectrum of RXFP1_(1–72)_ showing chemical shift effects on titration with EL1^(475–486)^/EL2-RXFP1 (**b**) chemical shift differences for wild-type RXFP_(1–72)_ (black), F54A (blue) and G41A/D42A (red) (**c**) wild-type RXFP_(1–72)_ (black), G45A/W46A (blue) and C40A^ins^ (red). Experiments were conducted at pH 6.8 and 25 °C.

Measuring the molecular interactions of H2 relaxin or LDLa-linker with full-length RXFP1 or its TMD is confounded by the multistep mode of activation^28^,^29^. While the LDLa module is indispensible for receptor activation^11^, the mutants of the LDLa-LRR linker presented here suggest critical roles of Asp42, Asn43, Gly45 and Trp46 in H2-mediated receptor activation (Table 1), with no direct involvement in H2 relaxin binding (Figs 2 and 6). This led us to investigate if the LDLa-LRR linker interacts with the exoloops of the TMD of RXFP1. To probe H2 relaxin and LDLa interactions with the exoloops of the TMD we have engineered a soluble protein scaffold where we graft exoloop-1 and -2 of the TMD onto the backbone of a thermostabilized version of the B1 immunoglobulin binding domain of streptococcal protein G (GB1) (ref. 14). Here we use a similar scaffold comprised of the entire exoloop-2 (Glu551 to Gln575) and the region of exoloop-1 (Ala475 to Gln486). This construct (EL1^(475–486)^/EL2-GB1) is similar to our previously reported EL1/EL2-GB1, except EL1 has been truncated which improves its expression and solubility. EL1^(475–486)^/EL2-GB1 maintains the disulfide between the C-terminal end of exoloop-1 and the centre of exoloop-2 that is essential for structure and function^30^. Titration of ^15^N-labelled RXFP1_(1–72)_ with EL1^(475–486)^/EL2-GB1 showed chemical shift changes for Gly41, Ser47 to Asp51, Ala55 and Lys59 to Thr61 (Fig. 7a,b) suggesting that the LDLa-LRR linker may interact with the ELs of the TMD. To test the specificity of side-chain interactions, the key linker mutations C40A^ins^, G41A/D42A, G45A/W46A and F50A RXFP1_(1–72)_ were selected for titration against EL1^(475–486)^/EL2-RXFP1. None of these mutants showed an interaction with EL1^(475–486)^/EL2-RXFP1 (Fig. 7b,c). However, the mutant, F54A-RXFP1_(1–72)_, which showed significant loss of H2 relaxin binding, but modest loss of activation, could still interact with EL1^(475–486)^/EL2-GB1 (Fig. 7b).

To show these interactions are dependent on the conformation of EL2, we prepared the scaffold protein substituting the two Cys residues with Ser, thus removing the disulfide bond between EL1 and EL2. This construct showed no chemical shift or line broadening in ^15^N-labelled RXFP1_(1–72)_ titrations supporting that the interaction is dependent on the conformation of EL2. Our previous functional studies on the full-length receptor showed that Trp479 of EL1 is proposed to be important for the low-affinity interaction of H2 relaxin, whereas Phe564 and Pro565 of EL2 are involved in LDLa-mediated activation^14^. We therefore prepared the equivalent mutations in EL1^(475–486)^/EL2-GB1. Titration of ^15^N-labelled RXFP1_(1–72)_ with the W479A mutation behaved similarly to EL1^(475–486)^/EL2-GB1 (Supplementary Fig. 7). However, titration of ^15^N-labelled RXFP1_(1–72)_ with either of the mutants F564A or P565A showed a marked reduction in chemical shift differences (Supplementary Fig. 7), although the interaction is not completely abolished as resonance broadening is still observed. Collectively, these data support the idea that residues of the LDLa-LRR linker make critical interactions with the exoloops of the TMD, which lead to activation of the receptor.

## Discussion

The current hypothesis of RXFP1 activation involves H2 relaxin binding with nanomolar affinity to the LRR domain via the arginine-binding cassette of the B-chain of H2 relaxin^31^ resulting in uncharacterized conformational changes of the ectodomain that drives binding of the LDLa module to the TMD to stabilize the active conformation^11^. Recently we showed that both the LDLa module and H2 relaxin are able to make contacts, albeit very weakly, with the exoloops of the TMD of RXFP1 which supports this interaction^14^. However, the conclusions we draw from the data presented here show that the 32-residue linker between the LDLa module and LRR domain binds with reasonable affinity to both H2 relaxin and EL2 of the TMD. Binding of H2 relaxin stabilizes and extends a helical conformation within the linker which we hypothesize is the conformational change that acts as the critical switch for LDLa-mediated receptor activation. The interacting surface of this helix is likely to be hydrophobic, due to significant loss of binding for alanine substitutions of Trp46, Phe50, Phe54 and Tyr58 in RXFP1_(1–72)_ and full-length RXFP1 (Table 1).

We propose that the high-affinity binding of H2 relaxin to the ectodomain of RXFP1 is therefore divided over two sites, the first involves the arginine cassette on the B-chain of H2 relaxin with the acidic and hydrophobic groups identified on a well-structured LRR domain^9^ and the second involving the A-chain of H2 relaxin with the region Trp46 to Gln63 of the LDLa-LRR linker. The titration data of RXFP1_(1–72)_ estimate the affinity for the LDLa-LRR linker/H2 relaxin interaction to be ∼200 μM, which predicts the affinity for the LRR domain to be ∼1 μM, where the latter appears consistent with the large loss of affinities observed for the RXFP1 mutants (Table 1). Notably the reported affinities in Table 1 for the G41A/D42A and G45A/W46A mutants (K_d_>20 nM) reflect the sensitivity of the assay (maximum H2 concentration 15 nM) and they clearly still bind H2 relaxin in the nanomolar range (Fig. 2a). Given the two binding sites are restricted to the same molecule, and taking some entropic losses into account, these dissociation constants are compatible with the expected affinity of ∼1 nM for H2 relaxin to RXFP1.

Currently solving the complex of H2 relaxin with RXFP1_(1–72)_ is complicated by the tendency of H2 relaxin to dimerize or aggregate at high concentrations which leads to exchange broadened NMR spectra^18^. To gain insight on the orientation of H2 relaxin to RXFP1_(1–72)_, we measured differential line broadening with Mn^2+^-DTPA-(A)-H2 at substoichiometric concentrations. As the paramagnetic label is attached to the N-terminus of Mn^2+^-DTPA-(A)-H2, the localization of the paramagnetic-induced broadening to the C-terminal end of the LDLa module and the first six residues of the linker indicates that the N-terminus of H2 relaxin is positioned near this site. Furthermore the titration and competition experiments with the H2 relaxin analogues confirm that the A-chain of H2 relaxin binds to the linker. The H2/I5-2 H2 chimera, which differs in sequence by two amino acid residues, His12 and Val13 (H2) to Thr12 and Asp13 (INSL5), could not bind to RXFP1_(1–72)_, suggesting these H2 relaxin A-chain residues may make specific contacts with the linker^22^. The importance of this interaction is supported by a previous observation whereby replacement of His–Val–Gly of H2 relaxin with Thr–Ser–Ile of the related peptide, human insulin, resulted in significant loss of activity^32^.

The profound reductions in H2 relaxin-stimulated cAMP activation observed for a number of linker mutations, including C40A^ins^, D42A, G45A and W46A, in the absence of any effect on activation by the allosteric agonist ML290, supports a key role for these residues in H2 relaxin-mediated activation. Importantly, mutations within the GDNNGW sequence in RXFP1_(1–72)_ abolished an interaction with the construct EL1^(475–486)^/EL2-GB1 suggesting that the LDLa-LRR linker interacts with EL2 in RXFP1. Mutation of the Phe564 or Pro565 of EL2, but not Trp479 of EL1, showed reduced binding to RXFP1_(1–72)_ supporting the importance of EL2 in this interaction and in agreement with the loss of activation for the equivalent mutations in the whole receptor^14^. Therefore we propose a second role for the linker residues, especially those within the GDNNGW sequence, which is to directly interact with the TMD exoloops.

**Figure 8:**
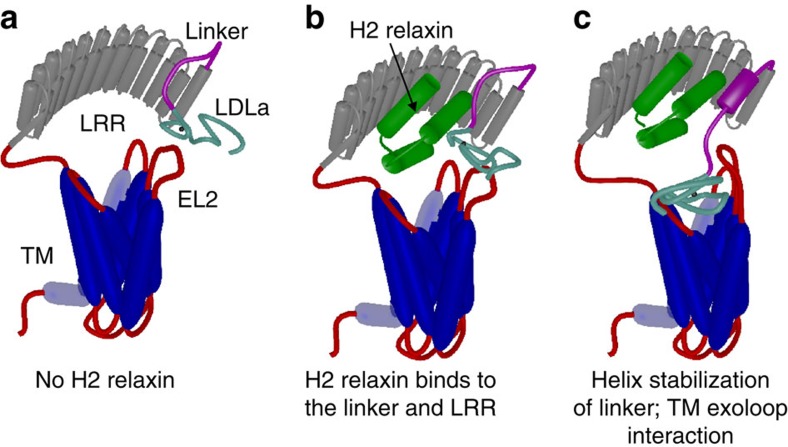
Mechanism of H2 relaxin binding to RXFP1. (**a**) Cartoon model of the domain structure of RXFP1. (**b**) H2 relaxin interacts and binds to the linker and LRR domain of RXFP1. (**c**) Binding of H2 relaxin stabilizes and extends a helix within the linker to orient and enable interactions of the LDLa module and residues within the linker to exoloop-2 of the TMD to facilitate receptor activation. The TMD (blue), LRR domain (grey), LRR-LDLa linker (magenta) and LDLa module (cyan) of RXFP1 are indicated. Additional loops are coloured red with exoloop-2 (EL2) of the TMD annotated. H2 relaxin is coloured green.

Consequently, we propose the functions of the residues of the linker as follows: residues within the sequence GDNNGW directly interact with EL2 of the TMD and together with the LDLa module^10^,^12^ are important in inducing the activated state of the receptor. Mutation of these residues results in loss of interaction with EL2 and a modest loss of H2 relaxin binding. While Gly45 and Trp46 may directly contact H2 relaxin, mutation of these residues shows that they also have important roles in maintaining the structure of the linker to bind H2 relaxin. Their mutation therefore has a combined effect on both H2 relaxin binding and receptor activation. Asp42 does not appear to have such a role in H2 relaxin binding, as the mutant G41A/D42A-RXFP1_(1–72)_ appears to bind H2 relaxin relatively normally, and the reasons for the significant loss of H2 relaxin affinity on mutation in RXFP1 are not clear. Phe50, Phe54 and Tyr58 are proposed to largely form the H2 relaxin-binding epitope. The mechanism we now propose is that H2 relaxin binds to the LRR via B-chain residues and to the linker via A-chain residues resulting in a high-affinity complex (Fig. 8). Binding of H2 relaxin simultaneously stabilizes and extends a helical conformational state of the linker. This conformational change positions residues of the linker, especially Gly41 to Trp46, and also residues of the N-terminal region of the LDLa module^10^,^12^ and H2 relaxin to form an interaction with the TMD resulting in receptor activation and the appropriate signalling pathways^10^,^12^,^14^,^28^,^29^.

RXFP1 is a Type C member of the LGR family of GPCRs^33^. The structure and mechanism of the Type A LGRs, such as the follicle-stimulating hormone receptor (FSHr), have been well studied^34^. The ectodomain of FSHr consists of a large LRR domain and a C-terminal hinge region that contains a sulfated tyrosine. On ligand binding there is little change to the structure of the LRR domain, whereas there is a significant change to the conformation of the hinge region. The sulfated tyrosine binds to a hydrophobic pocket on the hormone lifting the hinge region and activating the receptor. Therefore there are clear similarities and differences between the Type A and C LGRs. Both use an LRR domain to capture the hormone; on capture in each case the hormone interacts with less-structurally defined elements of the ectodomain, although Type A, for example FSHr, with a hinge region C-terminal to the LRR domain, and Type C, for example, RXFP1, with a linker region N-terminal to the LRR domain. This interaction for Type A LGRs results in activation by relieving an inhibited state of the receptor that involves a conformational change of the hinge region. However, and in contrast, our data suggest that for RXFP1 the LDLa module and LDLa-LRR linker, on hormone binding, reorient and bind to stabilize and activate the receptor.

Considering the efficacy of relaxin as an emerging pharmaceutical in the treatment of acute heart failure^35^, our study has discovered and characterized for the first time a unique auxiliary binding site of H2 relaxin in the linker region of RXFP1 and new insights into the mechanism of activation. This knowledge paves a clear path for the development of small molecules to target this discrete binding site of the receptor and these could be potential leads as therapeutic agents against this disease.

## Methods

### Site-directed mutagenesis

Single and double point mutations were introduced into wild-type RXFP1 inserted in a pcDNA3.1^TM^/Zeo+ AmpR expression vector with N-terminal FLAG tag and bovine prolactin signal sequence using a QuickChange protocol with PrimeStar polymerase (Takara Clontech) according to manufacturer's instructions. Mutagenesis primers were designed according to ref. 36 such that they contained a short, ∼15 base-pair overlapping region over the codon to be mutated, and non-overlapping sequences extending in the 3′ direction of both forward and reverse primers (Supplementary Table 1) to give a final T_m_ of ∼60 °C.

### Receptor expression in HEK293T cells

HEK293T cells (ATCC #CRL-1573; American Type Tissue Culture Collection) were used for receptor expression, and were grown in Dulbecco's modified eagle medium supplemented with 10% foetal bovine serum, 1% l-glutamine and 1% penicillin/streptomycin in incubators maintained at 37 °C with 5% CO_2_ and 85% humidity. Transient transfections were achieved using lipofectAMINE 2000 (Invitrogen) according to the manufacturer's instructions.

### Cell surface expression assays

The presence of mutant receptors at the surface of cells was gauged by virtue of the FLAG epitope present on their N-termini using a plate-based ELISA assay^37^. HEK293T cells were seeded at 2.0 × 10^5^ cells per well in 24-well plates (Costar) pre-coated with poly(l-lysine) and transfected with 1 μg per well of plasmid DNA. After a further 24 h of growth, cells were washed in TBS/CaCl_2_ (50 mM Tris pH 7.4, 150 mM NaCl and 1 mM CaCl_2_) and fixed with 3.7% formaldehyde in TBS/CaCl_2_ for 20 min. Two washes followed and then incubation for 45 min at room temperature with 1% bovine serum albumin (BSA) in TBS/CaCl_2_ to block non-specific binding. The cells were then incubated with 10 μg ml^−1^ of anti-FLAG M1 monoclonal antibody (Sigma-Aldrich) in TBS/CaCl_2_ for 2 h at room temperature. Cells were washed twice more before a 15-min reblock in 1% BSA/TBS/CaCl_2_; then incubated for 1 h with 2 μg ml^−1^ goat anti-mouse Alexa Fluor 488 suspended in 1% BSA/TBS/CaCl_2_. Cells were then washed thrice and stored frozen at −80 °C overnight. Finally cells were thawed and lysed by incubating for 30 min with 200 μl per well of lysis buffer (50 mM Tris pH 7.4, 150 mM NaCl, 1 mM EDTA, 0.25% Triton X-100) at room temperature with shaking, and scraped and transferred to black 96-well plates (180 μl in each well; Costar) to be read on an Omega POLARstar plate reader (BMG labtech) with excitation at 490 nm and emission at 520 nm. Non-specific background was determined using cells transfected with empty vector and mutant receptor expression was expressed as the percentage of the wild-type receptor expression. Data are mean±s.e.m. from at least three independent experiments each performed in triplicate. Pooled data were analysed in GraphPad PRISM 6 using one-way analysis of variance and uncorrected Fisher's least square difference multiple comparison test.

### Eu^3+^-labelled H2 relaxin-binding assays

The affinity of mutant RXFP1 receptors was compared with wild-type RXFP1 using Europium-labelled H2 relaxin (Eu-H2) saturation binding assays^19^. HEK293T cells were seeded at 2.5 × 10^4^ cells per well in 96-well poly(l-lysine) pre-coated View plates (PerkinElmer). Cells in each well were transfected with the addition of 0.25 μg of receptor plasmid. The following day, cells were washed with PBS (40 mM Na_2_HPO_4_, 1.5 mM KH_2_PO_4_ and 150 mM NaCl) and incubated at room temperature for 1 h with increasing concentrations of Europium-labelled H2 relaxin in the presence or absence of an excess of unlabelled H2 relaxin (1 μM), all made up in relaxin receptor binding buffer (20 mM HEPES, pH 7.5, 1.5 mM CaCl_2_, 50 mM NaCl and 0.01% NaN_3_) with 1% BSA. Cells were subsequently washed with PBS; to each well 100 μl Delfia Enhancement solution (PerkinElmer) was added and the plate shaken for 20–30 min before being read on the Omega POLARstar plate reader using time-resolved fluorescence and an excitation wavelength of 340 nm and emission of 614 nm. The data were analysed using GraphPad PRISM 6 and presented as mean fluorescent specific binding±s.e.m. and are representative of at least three independent experiments with triplicate determinations within each assay. Individual experiments were analysed by nonlinear regression one-site binding curves and resulting *K*_d_ values subjected to one-way analysis of variance and uncorrected Fisher's least square difference comparison test.

### cAMP activity assays

cAMP activity in response to H2 relaxin or ML290 was measured using a pCRE β-galactosidase reporter construct that was co-transfected with receptors in 1:1 proportion. Cells were seeded at 2.5 × 10^4^ cells per well into 96-well cell-bind plates (Corning)^38^. Eighteen hours after transfection, cells were stimulated with increasing concentrations of H2 relaxin or ML290 for 6 h at 37 °C. Media was aspirated and plates stored frozen at −80 °C overnight. ML290 dilutions were prepared in dimethyl sulphoxide (DMSO) and added to minimal media (Dulbecco's modified eagle medium containing 0.5% foetal bovine serum, 1% penicillin/streptomycin, 1% l-glutamine) such that wells contained 1% final DMSO concentration. H2 relaxin dilutions were prepared in minimal media containing 1% DMSO. Positive and negative control stimulations were achieved by incubation with 5 μM Forskolin and minimal media/1% DMSO, respectively. Plates were developed by 10 min of incubation in 25 μl of lysis buffer 1 (100 mM Na_2_HPO_4_, pH 8.0, 0.2 mM MgSO_4_ 0.01 mM MnCl_2_); a further 10 min of incubation in 100 μl of buffer 2 (100 mM Na_2_HPO_4_, pH 8.0, 2 mM MgSO_4_, 0.1 mM MnCl_2_, 0.5% Triton X-100, 40 mM β-mercaptoethanol); then to each well 25 μl of the β-galactosidase substrate, chlorophenol red-β-D-galactopyranoside (Roche) was added. All incubation steps were conducted with orbital shaking. Color-change was monitored and readings were taken on a Benchmark Plus Microplate Reader (Bio-Rad) at 570 nm. A minimum of three separate experiments were performed, each of them in triplicate, and data were pooled and presented as percentages of the maximum response induced by 5 μM Forskolin±s.e.m. A nonlinear regression sigmoidal dose-response curve was then fitted using GraphPad PRISM and resulting pEC_50_ and maximum response (*E*_max_) values were subjected to one-way analysis of variance and uncorrected Fisher's least square difference comparison test.

### Cloning of GB1-RXFP1_(1–72)_

The DNA sequence encoding the amino residues Gln1-Glu72 was amplified by PCR and inserted into the vector pGEV2, a thrombin-cleavable GB1 fusion expression vector using BamHI and XhoI restriction sites. Site-specific mutants were prepared using PrimeStar DNA Taq Polymerase (Takara Clonetech) following the manufacturer's instructions. Complimentary forward and reverse primers (Supplementary Table 1) were designed to introduce mutations into the pGEV-RXFP1_(1–72)_ plasmid. PCR products were incubated with 1.5 μl Dpn 1 (Promega) for 2 h before transformation into competent DH5α *E. coli* cells. DNA was isolated from single colony and the mutation verified by sequencing.

### Expression and purification of RXFP1_(1–72)_

The expression and purification of RXFP1_(1–72)_ was based on the method previously described for the LDLa module (RXFP1_(1–40)_) with minor modifications^39^. Briefly, protein was expressed into BL21 (DE3) *trxB* (Novagen) using autoinduction where protein expression via the T7 promoter is regulated by levels of lactose/glucose in the medium^40^. For uniform ^15^N isotopic labelling, GB1-RXFP1_(1–72)_ was expressed in N5052 minimal medium^40^ using ^15^NH_4_Cl (Sigma-Aldrich). For ^13^C,^15^N labelling, cells were grown in a 1 l Braun Biostat fermenter supplemented with ^15^NH_4_Cl and D-[^13^C] glucose as the sole nitrogen and carbon sources, following the protocol of Cai *et al.*^41^ Cells were harvested, pelleted and stored at −20 °C.

Cell pellets were resuspended in 50 mM Tris, 150 mM NaCl, pH 7.4 in the presence 5 mM EDTA and 1 mM phenylmethylsulfonyl fluoride (PMSF) and lysed using an Avestin EmulsiFlex C3 cell crusher. Cell debris was removed by centrifugation at 13,000*g*, 4 °C, for 40 min, and the soluble fraction passed through a 0.45-μM filter (Sartorius). The fusion protein was purified using IgG Sepharose 6 Fast Flow beads eluting with 50 mM acetic acid, pH 3.4. Eluted protein was buffer exchanged into 50 mM Tris-HCl, 150 mM NaCl, pH 8.5 via dialysis; concentrated to 100–300 μg ml^−1^ in refolding buffer (3 mM GSH, 0.3 mM GSSG, 50 mM Tris-HCl, 150 mM NaCl, 5 mM CaCl_2_, pH 8.5) and incubated overnight at 4 °C with stirring. The GB1 fusion was cleaved from the oxidized RXFP1_(1–72)_ by incubating overnight with thrombin (10 units per mg protein). The cleaved protein was further purified by gradient reversed phase high-performance liquid chromatography (RP-HPLC) (buffer A 0.1% trifluoro-acetic acid, buffer B 100% acetonitrile with 0.1% trifluoro-acetic acid) using an Agilent Zorbax 300SB-C18 column. Cleaved RXFP1_(1–72)_ elutes at 40 % buffer B. Collected fractions were lyophilized and stored at −20 °C.

### Cloning of EL1^(475–486)^/EL2-GB1

The DNA sequence encoding the EL1^(475–486)^/EL2-GB1 (to include residues Ala475 to Gln486 of EL1 and full-length EL2 (Glu551 to Gln575) of RXFP1) were sub-cloned into the expression vector pET15b (Novagen Inc.) using the NdeI and PstI restriction sites. The site-directed mutants W479A, F564A, P565A and the EL1^(475–486)^/EL2-GB1cs (where the cysteines are mutated to serine) were generated using PrimeStar DNA *Taq* polymerase (Takara Clontech) following the manufacturer's protocol. Primer sequences are in Supplementary Table 2. The reaction mixture was then subjected to Dpn1 treatment for 1.5 h before transformation into competent top10 *E. coli* cells. DNA extraction from the selected colonies was performed using Promega Wizard Plus SV mini prep kit and the mutations were verified via sequencing.

### Expression and purification of EL1^(475–486)^/EL2-GB1

These scaffold proteins were expressed with a thrombin-cleavable N-terminal His_6_ tag in BL21 (DE3) *E. coli* induced with 0.5 mM isopropyl -D-1-thiogalactopyranoside at OD_600_ of 0.6 at 16 °C for 16 h. Cells were harvested, pelleted and stored at −20 °C. Cells were lysed using an Avestin EmulsiFlex C3 cell crusher and centrifuged at 4 °C, 13,000*g* for 40 min to remove insoluble cell debris. Scaffold proteins were purified from the soluble fraction by affinity chromatography over Talon Superflow resin (Takara Clontech), which was equilibrated with 20 mM Tris-HCl, 150 mM NaCl, and 5 mM imidazole at pH 7.4 and were eluted with 400 mM imidazole in 20 mM Tris-HCl, 150 mM NaCl at pH 7.4. The His_6_ fusion partner was cleaved by thrombin enzyme (Sigma-Aldrich). Scaffold proteins were further purified using a HiLoad 16/60 Superdex 75 prep grade column (GE Healthcare), where it elutes as a single monomeric peak in 20 mM Tris-HCl, 150 mM NaCl at pH 7.4. Collected fractions were pooled, buffer exchanged to 50 mM Imidazole, 10 mM CaCl_2_ and pH 6.8 for recording NMR experiments.

### Solid-phase peptide synthesis

The individual A- and B-chain peptides of H2 relaxin were synthesized using Fmoc chemistry with orthogonal protecting groups for cysteine residues. The DTPA-tetra (*tert*-butyl (tBu) ester) chelator (3 eq, 0.3 mmol; Macrocyclic, Dallas, USA) was coupled to the N-terminus of solid-phase-bound A-chain peptide. The peptides (A- and B-chain) were cleaved from the solid support using a cocktail of trifluoro-acetic acid: 3,6-dioxa-1,8-octanedithiol: H_2_O: triisopropylsilane (94%: 2.5%: 2.5%: 1%), 20 ml for 120 min. Three disulfide bonds were formed in solution regioselectively^19^. Briefly, the first intra-A-chain disulfide bond was formed by 2,2′-dithiodipyridine-mediated air oxidation. The A-chain was activated by substituting the tBu protecting group of Cys11 with a S-Pyridyl group. This activated A-chain was then combined with the B-chain bearing free thiol at Cys10 to form a second disulfide bond. Finally, the third disulfide bond was formed by simultaneous removal of acetamidomethyl protecting groups using iodine. The DTPA-(A)-H2 relaxin was analysed and purified by RP-HPLC, and characterized by matrix assisted laser desorption/ionization time of flight (MALDI-TOF) mass spectrometry ([M+H]^+^_obs_ 6354.914).

### Mn^2+^-DTPA-(A)-H2 preparation

The purified peptide, DTPA-(A)-H2 relaxin, was dissolved in aqueous triethylammonium acetate buffer (0.3 mg of peptide per ml of solvent), pH 6.8. Manganese chloride (2 eq) was then added to the peptide solution and stirred for 15 min. The formation of complex between manganese and DTPA chelator was monitored by RP-HPLC using 20 mM triethylammonium acetate buffer (buffer A) and 90% ACN+10% buffer A (buffer B) with gradient 15–45% buffer B in 30 min. The starting peptide (DTPA-(A)-H2 relaxin) eluted at 22.615 min, whereas the final product (Mn^2+^-DTPA-(A)-H2) eluted at 24.161 min in HPLC analysis. The molecular weight was determined by MALDI-TOF mass spectrometry ([M+H]^+^_obs_.=6409.062).

### NMR spectroscopy

All RXFP1_(1–72)_ protein samples for nuclear magnetic resonance (NMR) experiments were prepared from lyophilized protein in 50 mM imidazole, with 10 mM CaCl_2_ at pH 6.8. Recombinant human gene-2 H2 relaxin peptide was kindly provided by Corthera. EL1^(475–486)^/EL2-GB1 samples were prepared as described above. Titrant and titrand were dialyzed in the same buffer in the same vessel. All NMR experiments were routinely collected at 25 °C on a 700-MHz Bruker Avance HDIII spectrometer equipped with triple resonance cryoprobe. Due to limited solubility of RXFP1_(1–72)_ protein, fractional deuteration^42^ was used to aid assignment of the ^1^H, ^15^N and ^13^C resonances of the linker residues at 100 μM without or with 500 μM H2 relaxin. Backbone assignments were made from 3D HNCACB, HNCOCACB, HNCO and HNCACO experiments. Spectra were processed using NMRPipe (ref. 43) and typically Fourier-transformed after applying Lorentz-to-Gauss window functions in the direct dimension and cosine bells in the indirect dimensions. NMR data were analysed using SPARKY (Goddard, T.D. and Kneller, D.G., University of California, San Francisco). Assignments of the mutant RXFP1_(1–72)_ proteins were determined from the assignments of wild-type RXFP1_(1–72)_.

The binding of H2 relaxin to RXFP1_(1–72)_ was monitored by acquiring 2D ^1^H-^15^N Heteronuclear Single Quantum Coherence (HSQC; 2,048 × 256 data points) for each titration point of increasing H2 relaxin: 25, 50, 100, 200, 400 and 500 μM against 50 μM RXFP1_(1–72)_. The volume of the NMR sample did not vary more than 10% over the course of titration. The weighted chemical shift perturbations (Δ*δ* p.p.m.) for ^15^N and the ^1^HN was computed using^44^ the equation Δ*δ* p.p.m.=((Δ^1^HN)^2^+(0.15 × Δ^15^N)^2^)^1/2^. Using peaks that were resolved, exhibited fast exchange, and shifted during the titration, equilibrium dissociation constants (*K*_d_) were estimated by nonlinear curve fitting, assuming two-site exchange, using xcrvfit 4.0.12 software (Boyko and Sykes, University of Alberta www.bionmr.ualberta.ca).

^15^N{^1^H}-NOE experiments^45^ (2,048 × 256 data points) were acquired with a saturation pulse of 4 s and an additional relaxation delay of 5 s. Sample conditions were 150 μM RXFP_(1–72)_ or mutants either without or with 450 μM H2 relaxin. Binding to Mn^2+^-DTPA-(A)-H2 to RXFP1_(1–72)_ and mutants was monitored by recording 2D ^1^H-^15^N HSQC (2,048 × 256 data points) for 0.2 μM Mn^2+^-DTPA-(A)-H2 against 50 μM RXFP1_(1–72)_. H2 relaxin and its truncates such as A-chain (9–24), H2/I5 chimera and Phe23Ala A-chain mutant H2 were titrated into a complex of 50 μM RXFP1_(1–72)_ and 0.2 μM Mn^2+^-DTPA-(A)-H2 with increasing concentrations ranging from 5 to 50 μM.

All scaffold EL1^(475–486)^/EL2-GB1 protein samples were prepared in 50 mM imidazole, with 10 mM CaCl_2_ at pH 6.8. The binding interaction of scaffold EL1^(475–486)^/EL2-GB1 protein to RXFP1_(1–72)_ was monitored by acquiring 2D ^1^H-^15^N HSQC for each titration point of increasing scaffold protein: 25, 50, 100, 200, 400, 500 and 1,000 μM against 50 μM RXFP1_(1–72)_. The volume of the NMR sample did not exceed 10% over the course of titration. The chemical shift mapping was computed as described above.

## Additional information

**Accession codes:** The assignments of the ^15^N, NH, ^13^Cα, Hα, ^13^Cβ and ^13^C′ of RXFP_(1–72)_ have been deposited at the BMRB with the accession code 26721.

**How to cite this article:** Sethi, A. *et al.* The complex binding mode of the peptide hormone H2 relaxin to its receptor RXFP1. *Nat. Commun.* 7:11344 doi: 10.1038/ncomms11344 (2016).

## Supplementary Material

Supplementary Information Supplementary Figures 1-7, Supplementary Tables 1-2.

## Figures and Tables

**Figure 1:**
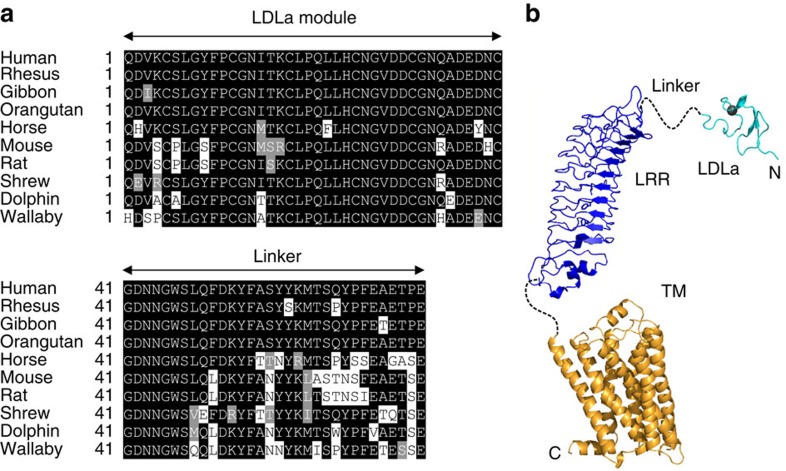
Sequence alignment of the LDLa-linker and domain structure of RXFP1. (**a**) Alignment of the sequences of the LDLa module and the 32-residue linker connecting to the LRR domain. Organisms shown human, rhesus (*Macaca mulatta*), Gibbon (*Nomascus leucogenys*), Orangutan (*Pongo abelii*), Horse (*Equus caballus*), Mouse (*Mus musculus*), Rat (*Rattus norvegicus*), Shrew (Tree Shrew; *Tupaia belangeri*), Dolphin (*Tursiops truncatus*) and Wallaby (*Macropus eugenii*). Sequences are from Genebank. (**b**) Model of the domain structure of RXFP1 showing the known structure of the N-terminal LDLa module (cyan) (pdb 2jm4) and homology models of the LRR (blue) and TM (orange) domains connected by linkers of unknown structure.

**Table 1:** Activity data for the mutant RXFP1 constructs used in this study compared with wild-type RXFP1.

**Receptor construct**	**Cell surface exp (% RXFP1)**	***K***_**d**_ **nM**	**H2 relaxin E**_**max**_	**H2 relaxin pEC**_**50**_	**ML290 E**_**max**_	**ML290 pEC**_**50**_
RXFP1	100±4.58	0.57±0.06 (4)	116.3±4.2 (6)	10.97±0.05 (6)	91.1±6.3 (5)	6.82±0.15 (5)
G41D42-AA	107.48±13.69 (4)	>20	106.8±46.67 (3)	7.26±0.32 (3)***	81.9±3.9 (3)	6.96±0.04 (3)
N43N44-AA	108.97±12.03 (4)	3.28±0.57 (5)***	118.90±12.81 (3)	8.29±0.63 (3)**	84.51±7.6 (3)	6.69±0.02 (3)
G45W46-AA	96.76±13.37 (4)	>20	—	<6 (3)	85.4±16.5 (4)	7.29±0.17 (4)*
G41A	130.5±8.05 (3)	0.95±0.13 (4)	104.7±1.65 (3)	11.13±0.1 (3)	ND	ND
D42A	106.27±8.18 (3)	20.7±3.5 (3)***	—	<6 (4)	ND	ND
N43A	81.78±8.45 (3)	2.55±0.55 (3)***	120.23±7.81 (4)	9.06±0.48 (4)***	ND	ND
N44A	83.7±6.88 (3)	0.81±0.17 (4)	92.16±6.98 (4)	11.0±0.1 (4)	ND	ND
G45A	118.64±5.04 (3)	19.1±3.07 (3)***	135.94±31.68 (4)	7.48±0.57 (4)****	ND	ND
W46A	112.30±5.90 (3)	12.26±1.36 (4)***	109.6±13.69 (3)	7.02±0.18 (3)****	ND	ND
S47L48-AA	76.95±4.47 (3)	0.43±0.01 (3)	120.9±10.9 (3)	11.07±0.17 (3)	84.7±8.1 (4)	6.48±0.11 (4)
F50A	101.09±7.03 (4)	6.48±0.76 (4)***	91.9±2.1 (4)*	9.45±0.07 (4)***	65.6±5.6 (6)**	6.60±0.15 (6)
Q49A/F50A	99.44±8.76 (3)	6.74±1.66 (4)***	98.2±8.8 (3)	9.17±0.04 (3)***	69.7±4.48 (5)*	6.87±0.03 (5)
D51A	89.77±8.97 (4)	0.73±0.11 (3)	96.5±4.0 (3)	11.01±0.56 (3)	79.4±3.8 (4)	6.67±0.11 (4)
K52A	90.25±15.21 (4)	ND	154.63±33.73 (5)	11.06±0.11	ND	ND
F54A	118.21±12.8 (3)	2.43±0.46 (5)***	104.89±10.48 (4)	10.23±0.13 (4)***	89.68±11.93 (4)	6.72±0.03 (4)
A55L	126.58±19.22 (4)	1.54±0.22 (4)*	123.59±8.51 (5)	10.75±0.17 (5)*	111.9±16.4 (3)	6.71±0.03 (3)
A55S	137.4±18.75 (4)**	0.80±0.09 (3)	154.43±39.77 (5)	11.39±0.13 (5)	ND	ND
Y57A	138.6±7.34 (5)**	0.81±0.22 (4)	126.7±8.46 (5)	10.93±0.10 (5)	85.5±6.4 (3)	6.76±0.04 (3)
Y58A	91.88±8.46 (3)	1.56±0.09 (4)***	89.14±7.93 (3)	10.88±0.09 (3)	108.3±10.4 (3)	6.78±0.01 (3)
F54A/Y58A	95.09±10.55 (3)	6.51±1.33 (5)***^,#,ф^	99.94±2.19 (5)	9.87±0.08 (5)***^,#,θ^	72.26±3.24 (4)*	6.60±0.08 (4)
M60A	93.79±16.66 (3)	1.08±0.21 (5)	118.2±6.51 (4)	11.20±0.03 (4)	104.8±11.05 (4)	6.73±0.04(4)
T61A	127.22±8.88 (5)*	0.69±0.08 (3)	142.58±15.97 (4)	11.15±0.17 (5)	ND	ND
F66A	81.6±7.45 (3)	ND	88.29±3.52 (3)	11.20±0.08	ND	ND
E67A	91.14±7.86 (3)	ND	141.74±27.08 (5)	11.13±0.13	ND	ND
						
*Insertion/deletion mutants*						
C40A^ins^	97.10±7.68 (4)	3.92±0.82 (3)***	No activity	No activity	94.1±7.2 (3)	6.77±0.03 (3)
F50A^ins^	135.36±12.81 (4)**	16.7±5.8 (4)***	93.62±5.34 (3)	9.41±0.13 (3)***	82.8±7.8 (3)	6.85±0.10 (3)
E67A^ins^	128.19±18.28 (3)	ND	110.15±6.43(3)	11.32±0.04 (3)	ND	ND
A68^Δ^	113.97±14.5 (4)	ND	93.78±3.19 (3)	11.18±0.02 (3)	ND	ND

ND, not determined.

*K*_d_ values from Eu-H2 relaxin saturation binding experiments; cell surface expression data from receptor expression ELISA experiments; pEC_50_ and maximum activity (*E*_max_) values for both H2 relaxin and ML290 stimulated cAMP activity assays. The number of replicates of each experiment for each individual construct are shown in parentheses with data reported as±s.e.m. Significance is calculated using one-way analysis of variance (ANOVA) and uncorrected Fisher's least squares difference multiple comparison test.

*P<0.05; **P≤0.01; ***P≤0.001; ****P≤0.0001 versus RXFP1.

^#^*P*<0.05 versus F54A, ^ф^*P*<0.05 versus Y58A, ^θ^*P*≤0.001 versus Y58A.
