# Supplementary material for: The complex binding mode of the peptide hormone H2 relaxin to its receptor RXFP1
Source: Nat Commun. 2016 Apr 18;7:11344. doi: 10.1038/ncomms11344 (PMC4837482; doi:10.1038/ncomms11344)
Supplement: Supplementary Information — Supplementary Figures 1-7, Supplementary Tables 1-2. [file ncomms11344-s1.pdf]

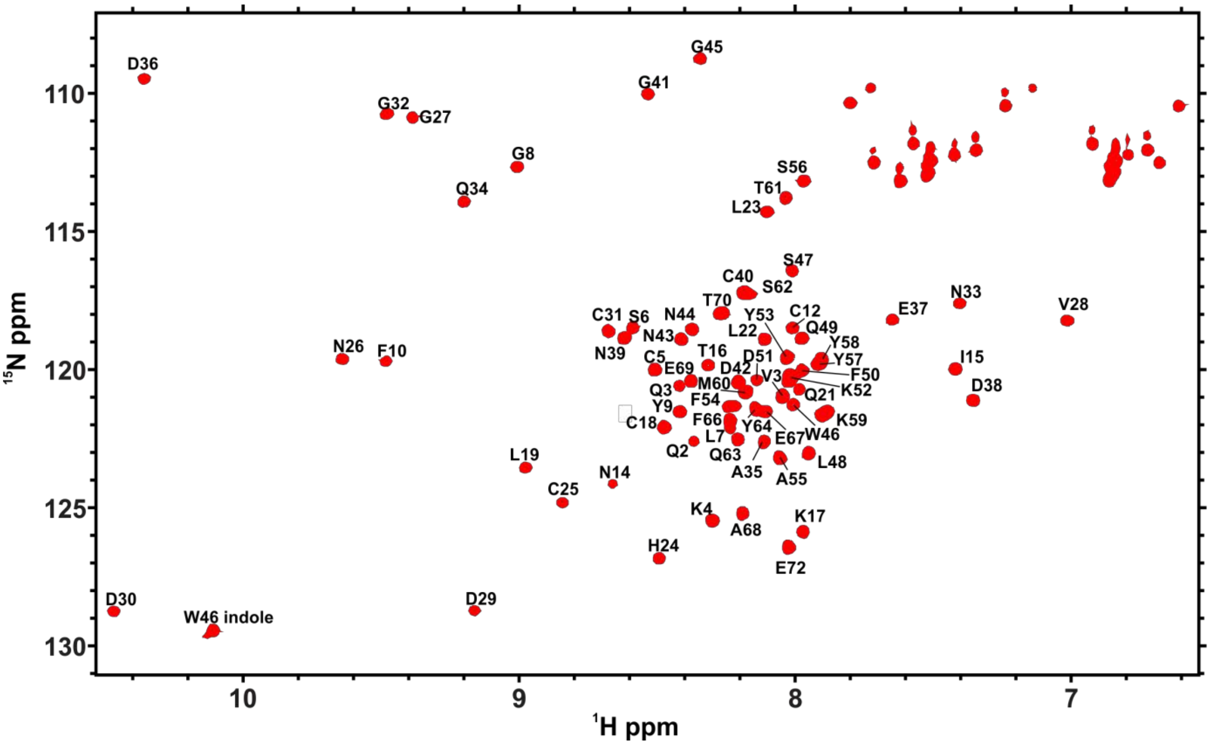

Supplementary Figure 1. ^{15}N-^{1}H HSQC of the LDLa module with the LDLa-LRR linker. All peptide amide signals are assigned and the indole NH signal of Trp46. Resonances in the upper right quadrant that are not assigned belong to Asn and Gln sidechains. Data were collected at pH 6.8 and 25 ºC.

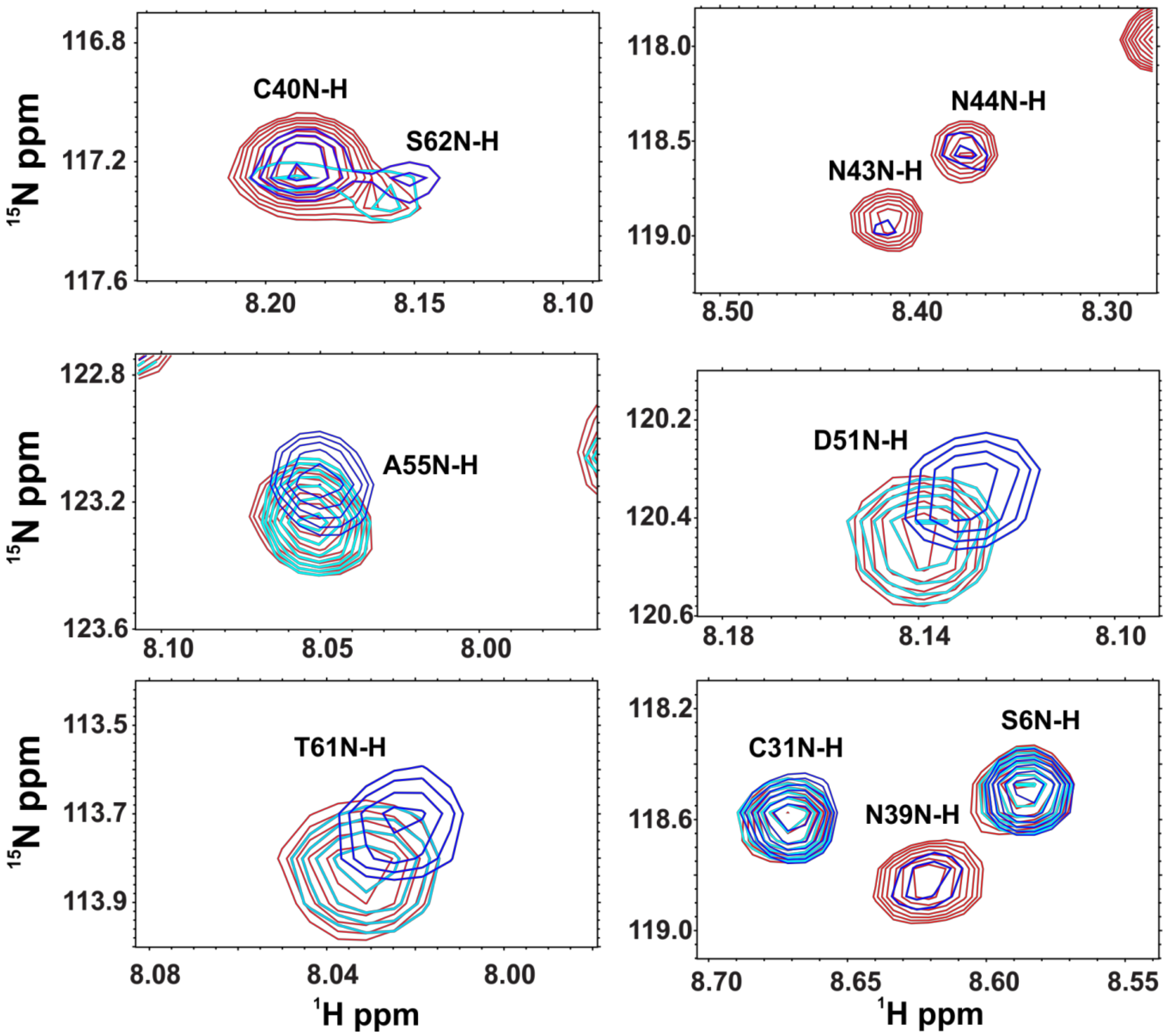

Supplementary Figure 2. Examples of intensity and chemical changes of 50 μM ^{15}N-labelled RXFP1$_{(1\text{-}72)}$ in the absence and presence of 0.2 μM Mn^{2+}-DTPA-(A)-H2 and competed with 50 μM H2 relaxin. In red are resonances of several NH groups in the absence of ligands. Upon addition of Mn^{2+}-DTPA-(A)-H2 some resonances completely broaden (Asn39, Asn43 and Asn44), while others are attenuated (Cys40) or remain unaffected (coloured in cyan). Addition of 50 μM H2 relaxin competes with Mn^{2+}-DTPA-(A)-H2 so that these broadened resonances regain intensity (coloured in blue). Resonances that belong to the H2 relaxin binding site (Fig. 2) shift on addition of H2 relaxin (for example, Asp51, Thr61 and Ser62). These residues while not broadened by Mn^{2+}-DTPA-(A)-H2, but are broadened by the stoichiometrically equivalent addition of H2 relaxin.

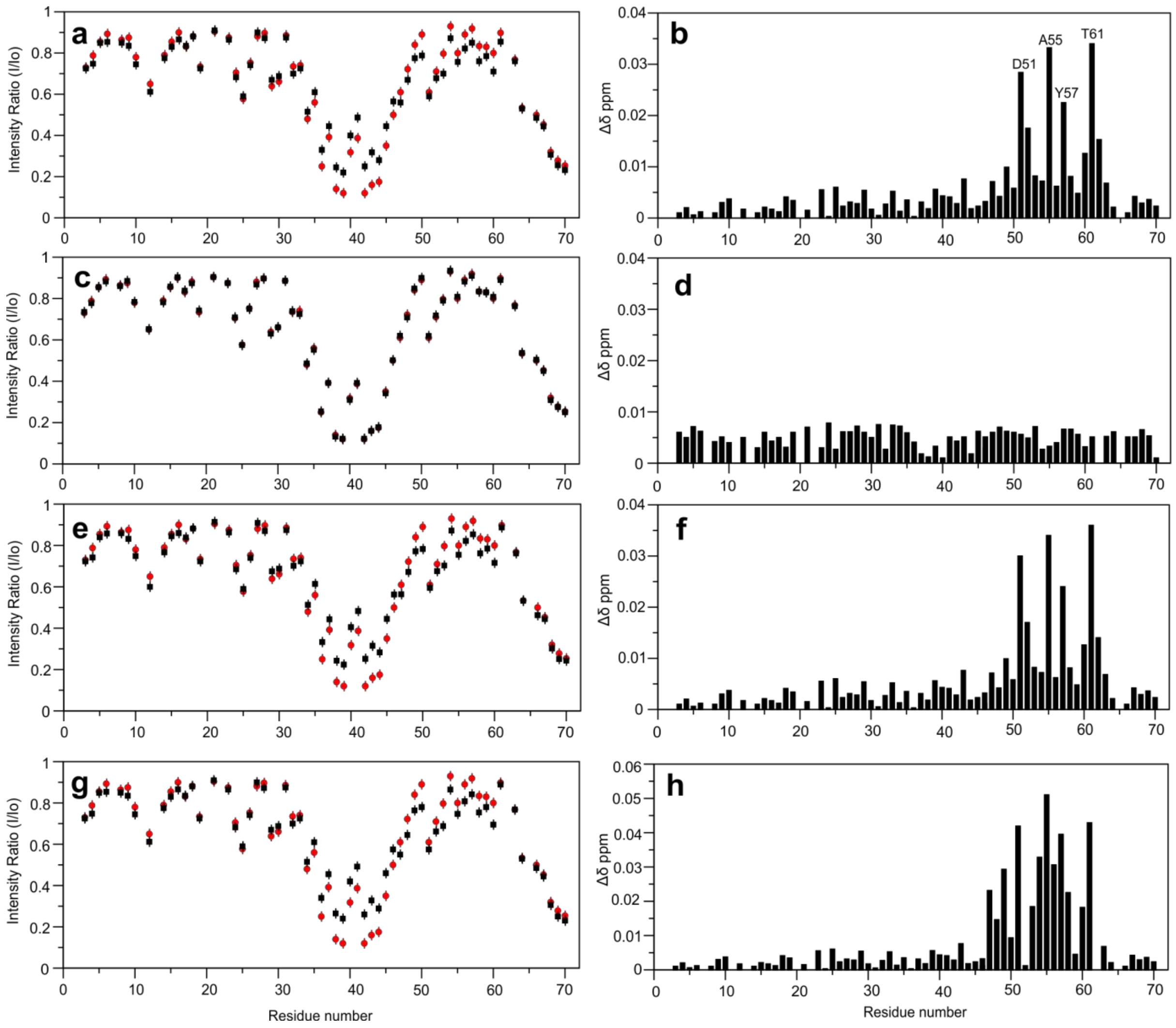

Supplementary Figure 3. Plots of intensity ratios and average chemical shift changes of 50 μM ^{15}N-labelled RXFP1$_{(1\text{-}72)}$ in the presence of 0.2 μM Mn^{2+}-DTPA-(A)-H2 and competed with 50 μM H2 relaxin or analogues. (**a**) and (**b**) H2 relaxin, (**c**) and (**d**) A-chain (F23A) H2 relaxin and (**e**) and (**f**) B-chain (B1-25), (**g**) and (**h**) B-chain (5-29). In (**a**), (**c**), (**e**) and (**g**) (red circles) represent ^{15}N-labelled RXFP1$_{(1\text{-}72)}$ in the presence of only Mn^{2+}-DTPA-(A)-H2; (black squares) indicate changes to the intensity ratio following addition of H2 relaxin or analogue. (**b**), (**d**), (**f**) and (**h**) show the change in average ^{1}H and ^{15}N chemical shifts after titration of the RXFP1$_{(1\text{-}72)}$ and Mn^{2+}-DTPA-(A)-H2 complex with one molar equivalent of H2 relaxin or analogue. Experiments were conducted at pH 6.8 and 25 ºC. Error bars represent the average estimated experimental noise for the respective NMR experiment.

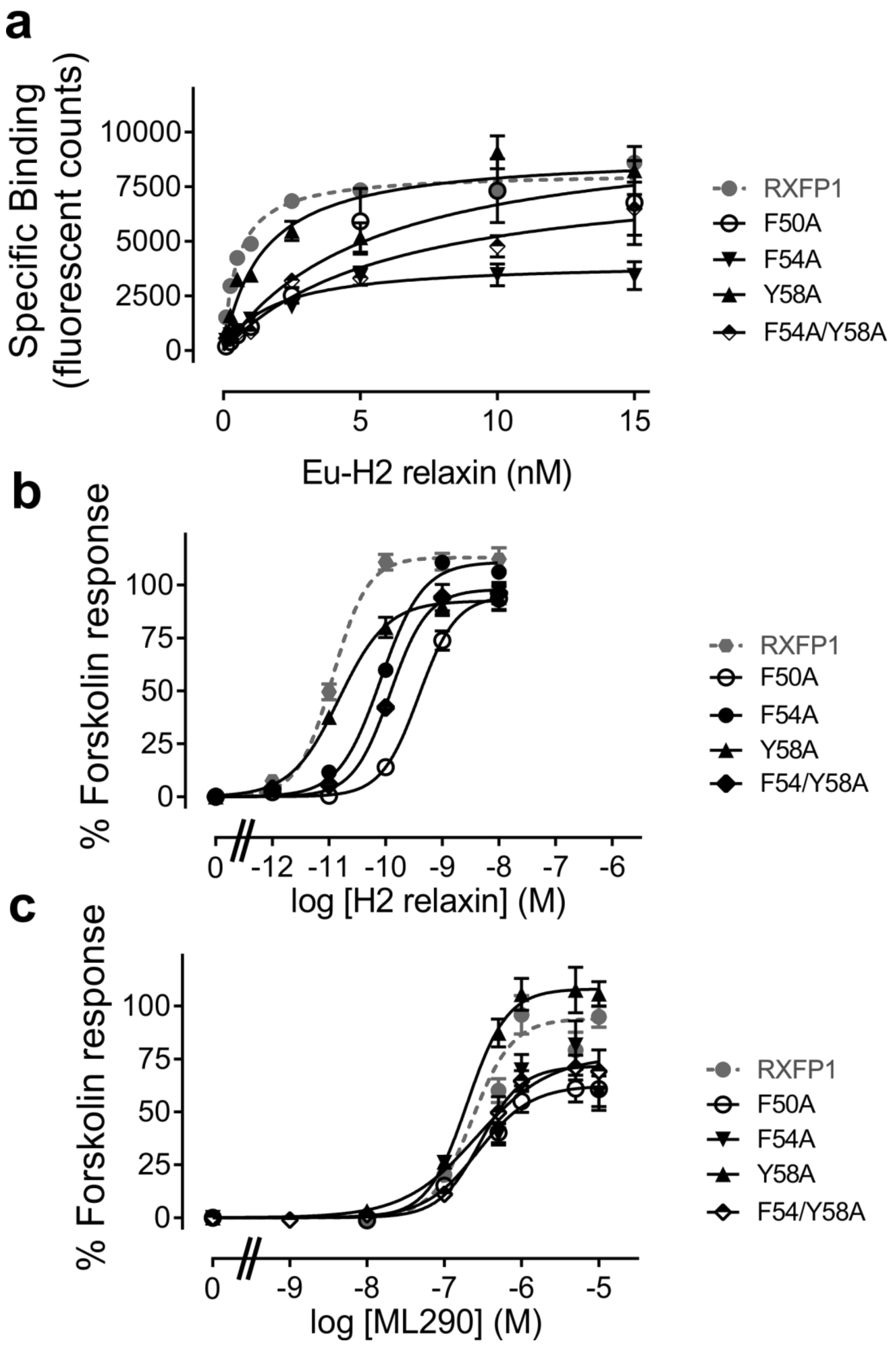

Supplementary Figure 4. H2 relaxin binding and activation of wild type and LDLa-linker mutants of RXFP1. (**a**) Competition binding using Eu-H2 relaxin. (**b**) H2 relaxin-induced cAMP responses (**c**) ML290-induced cAMP responses. Symbols represent mean values ± S.E.M from triplicate values in a minimum of three independent experiments.

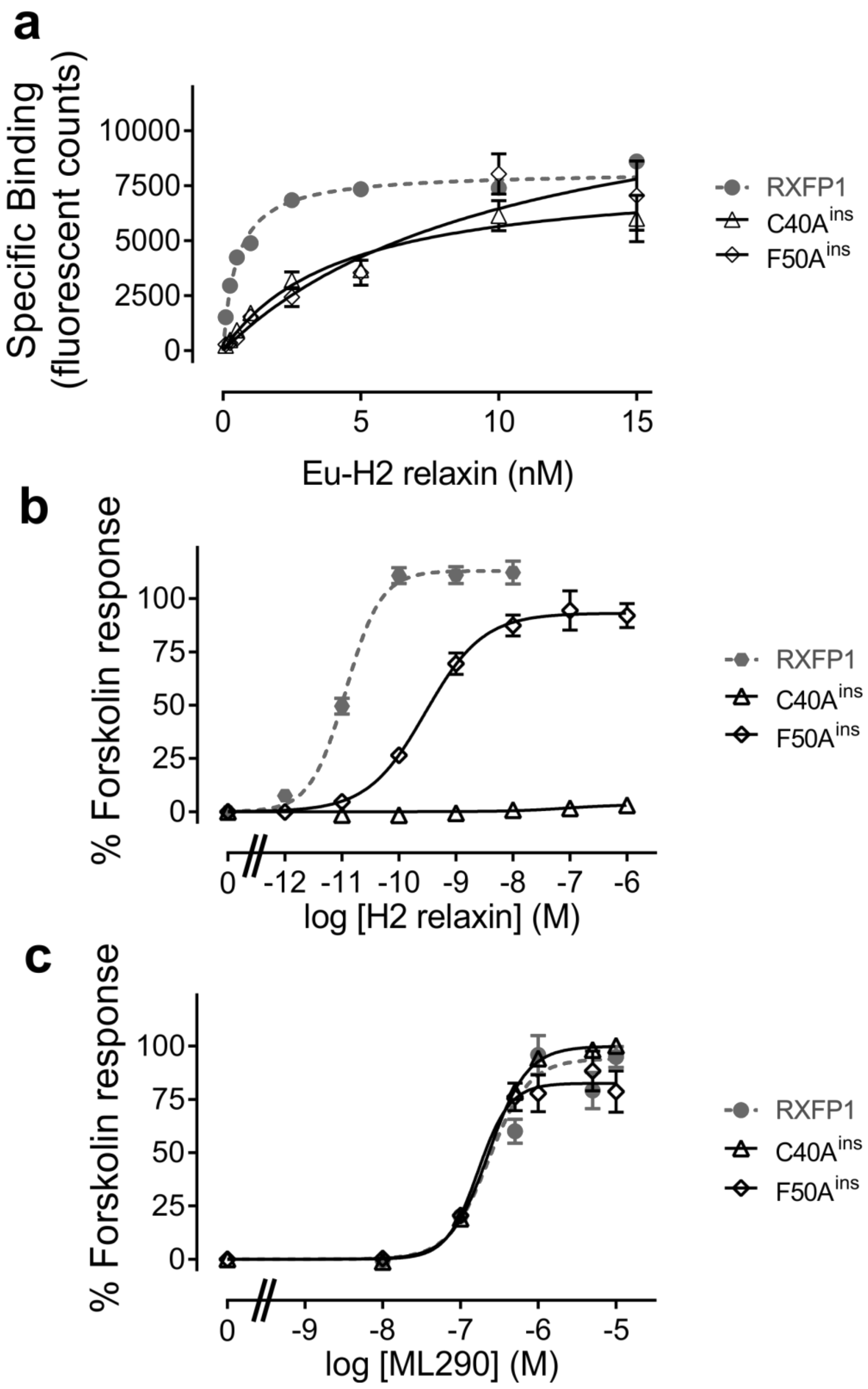

Supplementary Figure 5. H2 relaxin binding and activation of wild type and LDLa-linker insertion mutants of RXFP1. (**a**) Competition binding using Eu-H2 relaxin (**b**) H2 relaxin-induced cAMP responses (**c**) ML290-induced cAMP responses. Symbols represent mean values ± S.E.M from triplicate values in a minimum of three independent experiments.

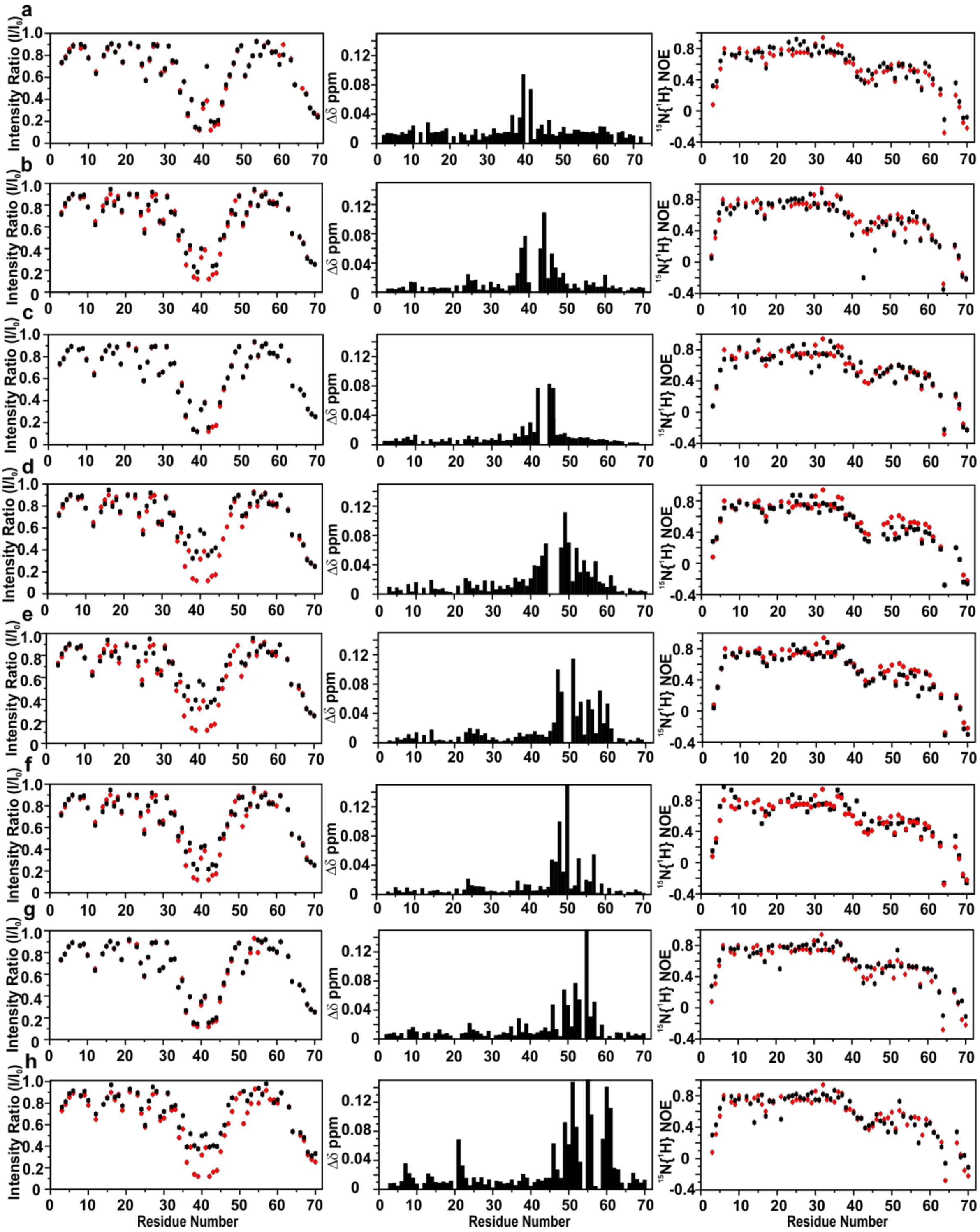

Supplementary Figure 6. Comparison of binding of Mn^{2+}-DTPA-(A)-H2, chemical shift differences and $^{15}N\{^{1}H\}$-NOEs of wild type and mutants of RXFP $_{(1\text{-}72)}$. The first column compares a titration of 50 µM mutant (black) to wild-type (red) LDLa-linker with 0.2 µM Mn^{2+}-DTPA-(A)-H2. The second column the average ^{1}H and ^{15}N chemical shift differences (Δδ) of mutant to wild-type protein and the third column the $^{15}N\{^{1}H\}$-NOE of mutant (black) to wild-type (red) LDLa-linker. Experiments were conducted at pH 6.8 and 25 ºC on (**a**) C40A^{ins}, (**b**) G41A/D42A, (**c**) N43A/N44A, (**d**) G45A/W46A, (**e**) F50A, (**f**) F50A^{ins}, (**g**) F54A, and (**h**) F54A/Y58A. Error bars represent the average estimated experimental noise for the respective NMR experiment.

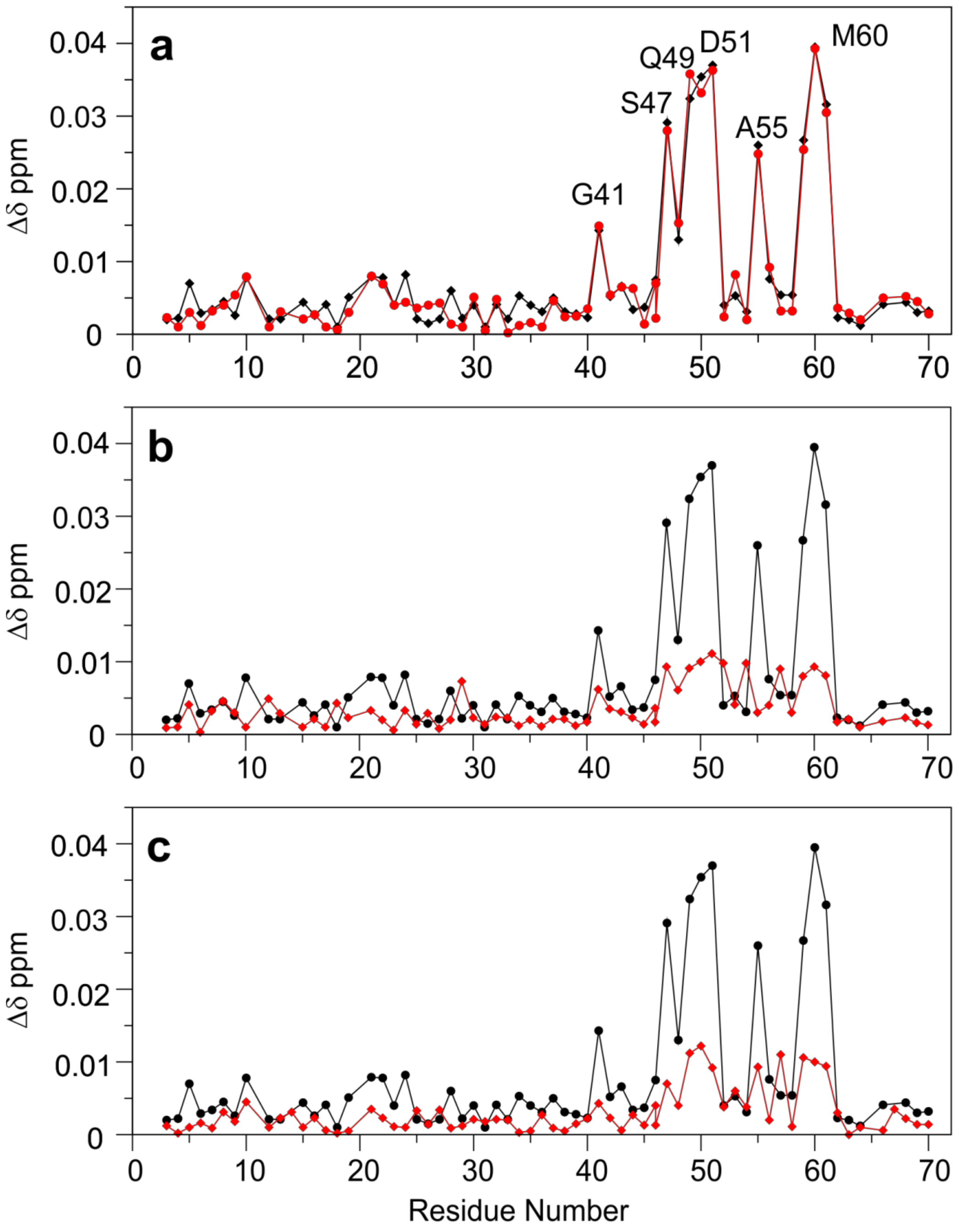

Supplementary Figure 7. Chemical shift differences following a titration of ^{15}N-labelled RXFP1$_{(1-72)}$ with the three mutants (a) Trp479 (b) Phe564 and (c) Pro565 of EL1$^{(475-486)}$/EL2-RXFP1. In each panel in black the average chemical shift changes of ^{1}H and ^{15}N for a titration of 50 µM ^{15}N-labelled RXFP1$_{(1-72)}$ with 20-times molar excess of wild type EL1$^{(475-486)}$/EL2-RXFP1 is shown. In red the results of a similar titration of ^{15}N-labelled RXFP1$_{(1-72)}$ with 20-times molar excess of each of the mutants is shown. Spectra were acquired at pH 6.8 and 25 ºC.

Supplementary Table 1. Primers used in site-directed mutagenesis of RXFP1 and RXFP1$_{(1\text{-}72)}$.

Mutant	Forward primer 5’ – 3’	Reverse primer 5’ – 3’
G41A/D42A	CTGTGCCGCCAACAATGGATGGTCTCTGCAATTTGACAAATATTTTG	GTTGGCGGCACAGTTGTCCTCATCGGCCTG
N43A/N44A	GACGCCGCCGGATGGTCTCTGCAATTTGACAAATATTTTGCC	CATCCGGCGGCGTCTCCACAGTTGTCCTCATCGG
G45A/W46A	CAATGCCGCCTCTCTGCAATTTGACAAATATTTTGCCAGTTACTAC	GAGAGGCGGCATTGTTGTCTCCACAGTTGTCCTCATCG
G41A	CTGTGCCGACAACAATGGATGGTCTCTGCAATTTGACAAATATTTTG	CTGGGCACAGTTGTCCTCATCGGCCTG
D42A	GGAGCCAACAATGGATGGTCTCTGCAATTTGACAAATATTTTG	GTTGGCTCCACAGTTGTCCTCATCGGCCTG
N43A	GACGCCAATGGATGGTCTCTGCAATTTGACAAATATTTTGCC	CCATTGGCGTCTCCACAGTTGTCCTCATCGG
N44A	CAACGCCGGATGGTCTCTGCAATTTGACAAATATTTTGCC	CATCCGGCGTTGTCTCCACAGTTGTCCTCATCGG
G45A	CAATGCCTGGTCTCTGCAATTTGACAAATATTTTGCCAGTTACTAC	CCAGGCATTGTTGTCTCCACAGTTGTCCTCATCG
W46A	GGAGCCTCTCTGCAATTTGACAAATATTTTGCCAGTTACTAC	GAGAGGCTCCATTGTTGTCTCCACAGTTGTCCTCATCG
S47A/L48A	GATGGGCTGCGCAATTTGACAAATATTTTGCCAGTTACTACAAAATGACTTC	CAAATTGCGCAGCCCATCCATTGTTGTCTCCACAGTTGTCCTCATC
F50A	GCAAGCTGACAAATATTTTGCCAGTTACTACAAAATGACTTCCC	AATATTTGTCAGCTTGCAGAGACCATCCATTGTTGTCTCC
Q49A/F50A	CTCTGGCAGCTGACAAATATTTTGCCAGTTACTACAAAATGACTTCC	ATATTTGTCAGCTGCCAGAGACCATCCATTGTTGTCTCCACAG
D51A	CAATTTGCCAAATATTTTGCCAGTTACTACAAAATGACTTCC	CAAAATATTTGGCAAATTGCAGAGACCATCCATTGTTGTCTC
K52A	GACGCATATTTTGCCAGTTACTACAAAATGACTTCCCAATATC	CAAAATATGCGTCAAATTGCAGAGACCATCCATTGTTGTC
F54A	CAAATATGCTGCCAGTTACTACAAAATGACTTCCCAATATCC	CTGGCAGCATATTTGTCAAATTGCAGAGACCATCCATTGTTG
A55L	CAAATATTTTCTCAGTTACTACAAAATGACTTCCCAATATCCTTTTGAG	GTAACTGAGAAAATATTTGTCAAATTGCAGAGACCATCCATTGTTGTC
A55S	CAAATATTTTTCCAGTTACTACAAAATGACTTCCCAATATCCTTTTGAG	GTAACTGGAAAAATATTTGTCAAATTGCAGAGACCATCCATTGTTGTC
Y57A	CAGTGCCTACAAAATGACTTCCCAATATCCTTTTGAGGCAG	GTAGGCACTGGCAAAATATTTGTCAAATTGCAGAGACCATC
Y58A	GCCAGTTAC**GCC**AAAATGACTTCCCAATATCCTTTTGAGGCAGAAACAC	CATTTT**GGC**GTAACTGGCAAAATATTTGTCAAATTGCAGAGACCATCCATTGTTG
F54A/Y58A	GTTACGCCAAAATGACTTCCCAATATCCTTTTGAGGCAG	CATTTTGGCGTAACTGGCAGCATATTTGTCAAATTGCAG
M60A	CTACAAAGCGACTTCCCAATATCCTTTTGAGGCAGAAACACC	GGGAAGTCGCTTTGTAGTAACTGGCAAAATATTTGTCAAATTGCAG
T61A	CAAAATGGCTTCCCAATATCCTTTTGAGGCAGAAACACC	GGAAGCCATTTTGTAGTAACTGGCAAAATATTTGTCAAATTGCAG
F66A	CCTGCTGAGGCAGAAACACCTGAATGTTTGGTC	CTCAGCAGGATATTGGGAAGTCATTTTGTAGTAACTGGC
E67A	CTTTTGCGGCAGAAACACCTGAATGTTTGGTCGG	CTGCCGCAAAAGGATATTGGGAAGTCATTTTGTAGTAACTGG
C40A^{INS}	CAACTGTGCGGGAGACAACAATGGATGGT CTC	GTCTCCCGCACAGTTGTCCTCATCGGCC
F50A^{INS}	GGTCTCTGCAATTTGCCGACAAATATTTTGCCAGTTACTACAAAATG	GGCAAATTGCAGAGACCATCCATTGTTGTCTCCACAGTTG
E67A^{INS}	CAATATCCTTTTGAGGCCGCAGAAACACCTGAATGTTTGG	GGCCTCAAAAGGATATTGGGAAGTCATTTTGTAGTAACTGG
A68$^{\Delta}$	CCTTTTGAGGAAACACCTGAATGTTTGGTCGGTTCTGTGCC	CAGGTGTTTCCTCAAAAGGATATTGGGAAGTCATTTTGTAGTAACTG

Supplementary Table 2: Primers used in site directed mutagenesis of EL1$^{(475\text{-}486)}$/EL2-GB1 construct.

Mutant	Forward primer 5'-3'	Reverse Primer 5'-3'
EL1$^{(475\text{-}486)}$/EL2-GB1cs	TACTCATTCTCAGGGCGGCGCACAG	GCCGCCCTGAGAATGAGTACTCTCC
W479A	GCGCAGCTGGCGATGGAGAGTACTCAT	CTCTCCATCGCCAGCTGCGCCATATG
F564A	GGAGTATGCGCCCCTCTTCATTCAGAAGA	ATGAAGAGGGGCGCATACTCCATTGGTG
P565A	GTATGCTTCGCTCTTCATTCAGAAGATAC	GAATGAAGAGCGAAGCATACTCCATTGG
